# A noncanonical role of glycolytic metabolites controlling the timing of mouse embryo segmentation

**DOI:** 10.1126/sciadv.adz9606

**Published:** 2025-09-19

**Authors:** Hidenobu Miyazawa, Jona Rada, Nicole Prior, Paul Gerald Layague Sanchez, Emilia Esposito, Daria Bunina, Charles Girardot, Judith Zaugg, Alexander Aulehla

**Affiliations:** ^1^ Developmental Biology Unit, European Molecular Biology Laboratory (EMBL), Meyerhofstraße 1, Heidelberg 69117, Germany.; ^2^ Structural and Computational Biology Unit, European Molecular Biology Laboratory (EMBL), Meyerhofstrasse 1, Heidelberg 69117, Germany.; ^3^ Genome Biology Unit, European Molecular Biology Laboratory (EMBL), Meyerhofstraße 1, Heidelberg 69117, Germany.

## Abstract

Studies on the impact of metabolism on cell fate decisions are seeing a renaissance. However, a key challenge remains to distinguish signaling functions of metabolism from its canonical bioenergetic and biosynthetic roles, which underlie cellular homeostasis. Here, we tackled this challenge using mouse embryonic axis segmentation as an experimental model. First, we found that energetically subminimal amounts of glucose can support ongoing segmentation clock activity, providing evidence that glycolysis exerts a signaling function. Using a dynamical systems approach based on entrainment, we identified fructose 1,6-bisphosphate (FBP) as the potential signaling metabolite. Functionally, we demonstrated that glycolytic flux/FBP control the segmentation clock period and Wnt signaling in an anticorrelated manner. Critically, we showed that the slow segmentation clock phenotype caused by elevated glycolysis is mediated by Wnt signaling rather than cellular bioenergetic and biosynthetic state. Combined, our results demonstrate a modular organization of metabolic functions, revealing a signaling module of glycolysis that can be decoupled from its canonical metabolic functions.

## Teaser

Glycolysis plays a noncanonical signaling role via Wnt signaling to control the timing of mouse embryo axis segmentation.

## INTRODUCTION

Central carbon metabolism affects gene expression and signal transduction via various mechanisms, such as epigenetic and protein posttranslational modifications. In addition, it exerts its canonical bioenergetic and biosynthetic functions by producing energy, reducing equivalents, and cellular building blocks to fuel biological processes (*1*–*7*). The widespread roles and importance of metabolism are being reemphasized and increasingly studied. However, it remains a key challenge to identify specific molecular mechanisms by which metabolism exerts its instructive roles due to its intertwined role in cellular homeostasis and signaling (*8*).

To overcome the challenge, one strategy is to finely tune metabolism within a physiological range in a targeted manner and then monitor its impact, for instance, at the level of signaling in a quantitative and dynamic manner. Here, we developed such an approach in the context of vertebrate embryo mesoderm segmentation, which represents an excellent entry point to tackle the role of metabolism. Previous studies had firmly established a functional role for glycolysis in the development of the presomitic mesoderm (PSM) into somites, the precursors for vertebrae and skeletal muscles (*9*–*11*). At the same time, it is well studied that the timing of somite segmentation is regulated by the segmentation clock, linked in all vertebrates studied to the oscillatory signaling activity of the Notch signaling pathway (*12*). Temporal periodicity of Notch signaling oscillations is translated into spatial periodicity of somites by integrating additional information encoded by graded signaling pathways such as Wnt, fibroblast growth factor (FGF), and retinoic acid signaling (*13*–*16*). In the mouse PSM, FGF and Wnt signaling pathways are also core components of the segmentation clock, exhibiting oscillatory activities coupled to Notch signaling oscillations (*14*, *17*, *18*). This highly complex network of interconnected signaling pathways can be dynamically perturbed and functionally studied by using a combination of quantitative live imaging and a dynamical systems approach. For instance, using microfluidics-based entrainment, we previously showed that the segmentation clock network can be efficiently controlled via external periodic pulses of Notch and Wnt signaling cues, achieving synchronization and tuning of signaling oscillation period (*14*, *19*).

When combined with tailored metabolic perturbations, this ability for quantitative live imaging, mouse genetics, and microfluidics-based entrainment offers a unique opportunity to tackle the central question of whether and how glycolysis exerts its signaling function during PSM segmentation. As stated above, it has previously been established that active glycolysis is essential for maintaining the segmentation clock oscillations (*9*). Moreover, studies revealed that changes in central carbon metabolism affect Wnt signaling (*9*–*11*, *20*) and the period of the segmentation clock (*21*). Yet, these studies could not resolve whether the observed effects reflect a bioenergetic/biosynthetic or signaling function of metabolism. Here, we reveal that glycolytic flux controls the timing of mouse embryo axis segmentation via glycolytic flux signaling, a module that we can dissociate from the canonical bioenergetic/biosynthetic functions commonly considered. Furthermore, we localize the glycolytic flux signal to specific metabolites around the phosphofructokinase reaction. Particularly, we show evidence that fructose 1,6-bisphosphate (FBP) can potently signal to and entrain the segmentation clock. Combined, our results demonstrate that this glycolytic flux–signaling module is mediated at least, in part, via Wnt signaling, revealing a glycolysis-FBP-Wnt signaling axis.

## RESULTS

### Disentangling signaling and bioenergetic roles of glycolysis in the segmentation clock oscillations

**Fig. 1.**
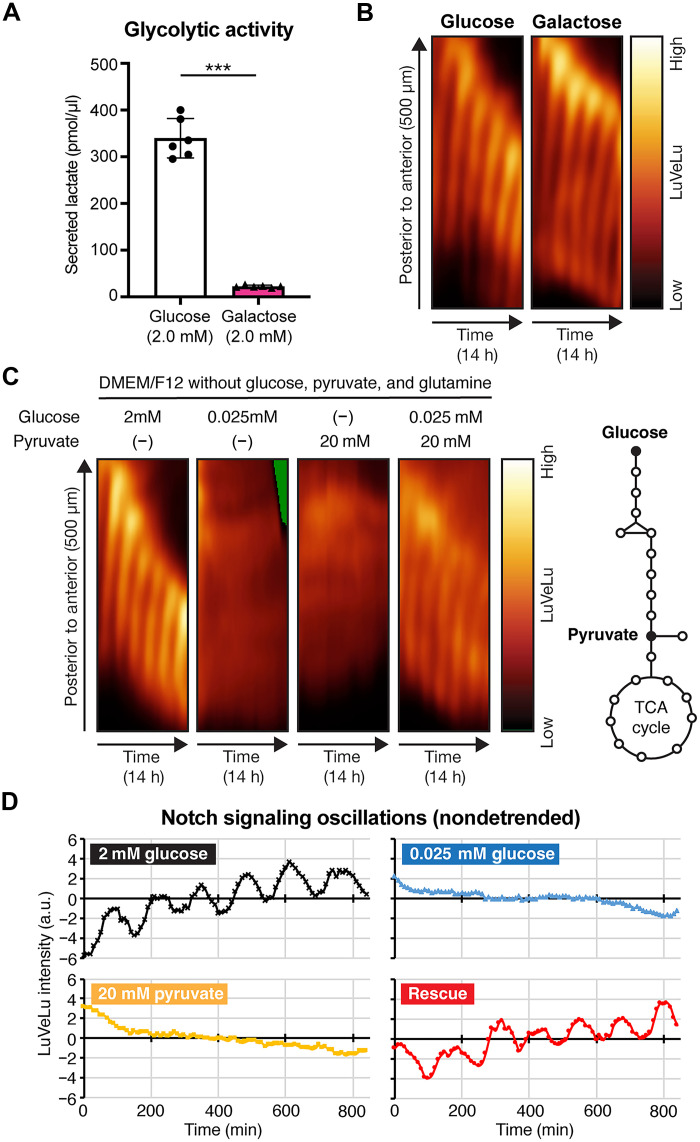
Rescuing the segmentation clock with a subminimal amount of glucose. (**A**) Lactate secretion was quantified as a proxy for glycolytic flux within PSM cells. The amount of lactate secreted from PSM explants during 12 hours of ex vivo culture was quantified. Welch’s *t*-test (***P < 0.001). Data are presented as means ± SD, and individual data points represent biological replicates. (**B** and **C**) Kymographs showing the dynamics of the Notch signaling reporter [=LuVeLu (*65*)] in wild-type PSM explants cultured in different culture conditions. In (B), PSM explants were cultured in DMEM/F12 (without glucose and pyruvate) supplemented with 2.0 mM glucose or 2.0 mM galactose, while, in (C), they were cultured in DMEM/F12 (without glucose, pyruvate, and glutamine) supplemented with either glucose (0.025 mM or 2.0 mM), pyruvate (20 mM), or both. h, hours. (**D**) Temporal profiles of LuVeLu intensities in the posterior PSM of samples shown in (C). The values were centered to the mean LuVeLu intensities over time for each sample. a.u., arbitrary unit.

To address whether glycolysis is required for signaling oscillations underlying the segmentation clock due to its bioenergetic/biosynthetic or signaling functions, we investigated the consequence of markedly reducing glycolytic flux using an established ex vivo culture system of mouse PSM explants. The control culture conditions mimic glucose concentrations the embryo is exposed to physiologically in utero [i.e., 2.0 mM; (*22*)] and build on refined culture settings, such as elevated oxygen concentrations, that are needed especially in static culture conditions to obtain faithful and timely development of mammalian embryos (*23*). Under these conditions, axis segmentation, clock oscillations, and elongation proceeded very reliably and comparable to in vivo (*24*). We first tested whether the segmentation clock and PSM patterning continue when galactose is provided as a primary carbon source instead of glucose. Galactose is metabolized through glycolysis at very low flux rates, hence forcing cells to rely on mitochondrial respiration for adenosine 5′-triphosphate (ATP) production (*25*). When glucose was replaced with galactose, glycolytic flux was reduced by 15-fold within PSM cells (Fig. 1A). Notably, despite the minimal glycolytic flux, we found qualitatively no changes in the segmentation clock oscillations or PSM segmentation (Fig. 1B and movie S1). As stated above, bypassing glycolysis entirely is not compatible with clock activity. Combined, our findings indicate that a minimal glycolytic flux is sufficient and required, which possibly reflects that this essential role is linked to a signaling function and not to a bioenergetic/biosynthetic role.

To further explore this possibility, we devised a “subminimal glucose rescue” experiment in mouse embryos, directly inspired by an experiment published in classic studies by Spratt (*26*) in chick embryos. In this rescue experiment, we first determined the minimal glucose concentration and, hence, minimal glycolytic flux that is sufficient to sustain PSM development and segmentation clock oscillations. Previously, we reported that PSM explants develop qualitatively normally even when glucose concentration is reduced to 0.5 mM (*9*, *11*).

When reduced to as low as 0.025 mM, the glucose concentration was subminimal and, thus, was no longer able to support the segmentation clock oscillations (Fig. 1, C and D). As previously shown, bypassing the entire glycolytic pathway by supplying pyruvate instead of glucose led to the expected immediate arrest of the segmentation clock activity (Fig. 1, C and D) (*9*). Notably, however, the combination of a subminimal amount of glucose (=0.025 mM) with pyruvate, two conditions that on their own both fail to support segmentation clock activity, led to a clear rescue of segmentation clock oscillations (Fig. 1, C and D; fig. S1A; and movie S2). We found that the subminimal amount of glucose had no significant impact on the cellular bioenergetic state, including the adenosine 5′-diphosphate (ADP)/ATP ratio and the oxidized to reduced from of nicotinamide adenine dinucleotide (NAD^+^/NADH) ratio (fig. S1, B and C). These findings provide evidence that the indispensable role of glycolysis is not linked to a bioenergetic effect; rather, we conclude that the minute, subminimal glucose amounts that rescue segmentation clock oscillations exert a signaling function. It is the first clear evidence directly supporting that glucose metabolism functions as a signal in this complex developmental process.

### Metabolic entrainment of the segmentation clock

To localize the signal within the glycolytic pathway, we used a dynamical systems approach on the basis of entrainment. Entrainment offers a quantitative and nondisruptive approach to reveal functional dependencies within a dynamical system. We had previously established microfluidics-based entrainment of the mouse embryo segmentation clock, using periodic pulses of signaling pathway modulators, such as a Notch signaling inhibitor and a Wnt signaling activator (*14*, *19*).

**Fig. 2.**
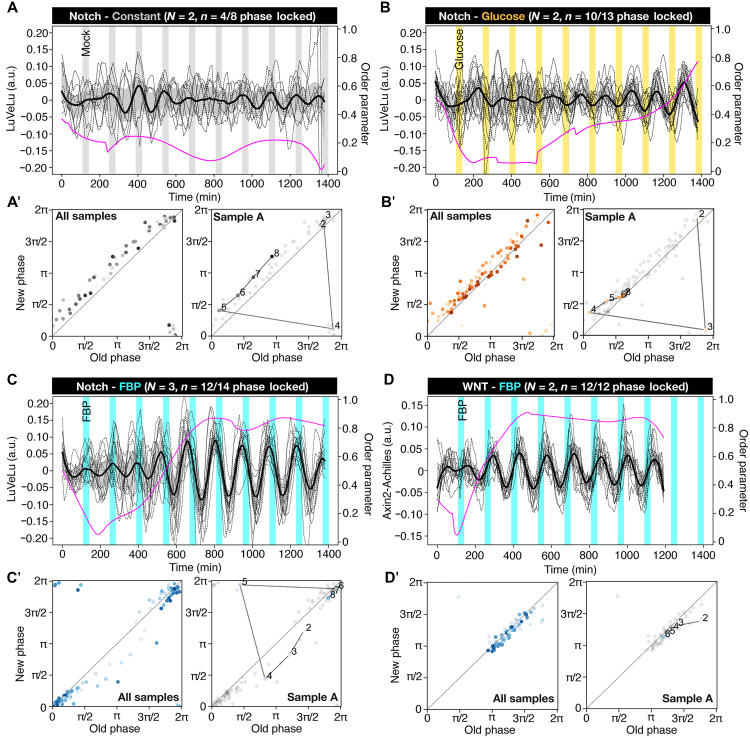
Metabolic entrainment of the segmentation clock. (**A** to **D**) Detrended (via sinc-filter detrending, cutoff period of 240 min) time series of LuVeLu [(A) to (C)] and Axin2-Achilles (D) intensity oscillations in wild-type PSM explants during metabolic entrainment (dashed lines, individual samples; bold black lines, median values; gray shades, the first to third quartile range; a.u., arbitrary unit). Changes in the first Kuramoto order parameter are shown in magenta. Samples were incubated either in a constant (i.e., 2.0 mM) glucose condition with periodic mock pulses (gray) (A) or alternating culture conditions [(B) to (D)] with a period of 140-min and a pulse length of 30-min [alternating between (B) 2.0 mM (white) and 0.5 mM (yellow) glucose conditions; [(C) and (D)] the medium with (cyan) or without (white) 20 mM FBP on top of 2.0 mM glucose]. To keep molarity of the medium at constant during experiments, nonmetabolizable glucose analog (i.e.,3-O-methyl-D-glucopyranose) was added to the medium when necessary. (**A′** to **D′**) Stroboscopic maps showing step-wise changes in the phase of LuVeLu [(A′) to (C′)] and Axin2-Achilles oscillations (D′) during metabolic entrainment. At each pulse of metabolic perturbations with glucose [(A′) and (B′)] or FBP [(C′) and (D′)], the phase of the oscillator (i.e., new phase) is plotted against its phase at the previous pulse (i.e., old phase). Darker dots represent later time points. Stroboscopic maps of a single representative sample are shown on the right (the numbers in the plots indicate the number of pulses).

Our reasoning was that if glycolytic metabolites act as signals to the segmentation clock, then we should be able to use metabolites to entrain the signaling oscillations of the segmentation clock. Although we perturb metabolism in a periodic manner, metabolism does not have to be oscillatory within PSM cells for metabolic entrainment to work. We first tested whether periodic changes in glucose levels can entrain the segmentation clock. Changes in glucose levels are mirrored at the level of glycolytic flux and several metabolite levels in PSM cells (*11*). We used microfluidics to implement periodic changes in glucose concentration during the culture of intact PSM explants and monitored segmentation clock dynamics using real-time imaging of a Notch signaling reporter. Notably, we found that periodic alternations of glucose levels are sufficient to entrain Notch signaling oscillations underlying the segmentation clock (Fig. 2, A, A′, B, and B′; and movie S3). We quantified entrainment based on phase-locking (Fig. 2, A′ and B′, and fig. S2C) and also using the first Kuramoto order parameter (Fig. 2, A and B), which effectively measures the synchronicity of signaling oscillations between samples.

We then tested several glycolytic metabolites for their ability to signal and entrain the segmentation clock. We especially focused on the sentinel metabolite FBP, which we previously revealed to function as a sentinel metabolite mirroring glycolytic flux within PSM cells (*11*). Excitingly, we found that periodic addition of FBP efficiently entrained Notch signaling oscillations, with a distinct phase locking compared to the glucose entrainment (Fig. 2, C and C′; fig. S2C; and movie S4). In contrast, periodic application of pyruvate, the end product of glycolysis that feeds into the TCA cycle, did not entrain the segmentation clock based on our quantitative criteria, i.e., lack of phase-locking and lack of increase of the first Kuramoto order parameter (fig. S2, A, A′, and C; and movie S5). The entrainment capacity of glucose and FBP, combined with the failure of pyruvate to elicit a similar effect, allowed us to localize the metabolic signal to a specific level of glycolysis, centered around the metabolite FBP, which is located in the committed part of glycolysis (*27*). This finding is in apparent contrast with recent work in embryonic stem cell models. In that in vitro context, it was reported that pyruvate exerted an effect on the segmentation clock period via effects on the cellular redox state (*21*). While we could confirm that pyruvate supplementation led to an increase in the NAD^+^/NADH ratio (fig. S2D) in our assays, such changes in cellular redox state did not correlate with entrainment of the segmentation clock. Hence, our results indicate that, in the mouse embryo PSM explant system that we used, which, in contrast to the stem cell culture approaches, does not rely on exogenous growth and signaling factors, cellular redox state is not essential in mediating the effect of glycolysis that we observed. Rather, our results point to a signaling role of glycolytic metabolites, a conclusion that we wanted to further challenge using a nonmetabolizable FBP analog.

To this end, we used the fructose analog 2,5-anhydromannitol (2,5-AM). 2,5-AM is converted to the FBP analog AM–1,6-bisphosphate, which, in turn, cannot be metabolized any further (*28*–*30*). We tested whether we can entrain the segmentation clock using 2,5-AM. Intriguingly, periodic application of 2,5-AM efficiently entrained Notch signaling oscillations (fig. S2, B, B′, and C; and movie S6). This finding is compatible with the conclusion that it is FBP itself that acts as a signal to the segmentation clock. One caveat, however, is our finding that at least the chronic addition of 2,5-AM also had an effect on glycolytic flux (fig. S2E). While we were not able to score such a flux-reduction effect with periodic 2,5-AM entrainment, it remains a possibility that 2,5-AM entrained the clock via periodic changes in glycolytic flux and, hence, via multiple metabolites.

We used metabolic entrainment to further disentangle the functional dependencies between glycolysis and signaling oscillations underlying the segmentation clock. We previously had shown that Wnt and Notch signaling oscillations are coupled within the segmentation clock network and that entrainment of Notch signaling oscillations eventually leads to entrainment of Wnt signaling oscillations with a time delay and vice versa (*14*). Thus, we next quantified the timing of FBP entrainment in regard to both Notch and Wnt signaling oscillations. Notably, we found that periodic FBP pulses first entrained Wnt signaling oscillations, while entrainment of Notch signaling oscillations followed with considerable time delay (Fig. 2, C and D; fig. S2F; and movie S7). Hence, this dynamic entrainment analysis provides strong evidence that glycolysis/FBP signals to the segmentation clock first via Wnt signaling.

It was previously reported that glycolysis affects Wnt signaling via changes in intracellular pH, proposed to affect acetylation of ß-catenin (*10*). However, in the mouse embryo PSM system that we studied, we could not find a correlation between glycolytic flux and acetylated ß-catenin levels (fig. S3). This is in agreement with our combined results that reveal a glycolysis-FBP-Wnt signaling axis, which can be decoupled from the bioenergetic and biosynthetic functions of the glycolytic pathway.

While we noticed that, during metabolic entrainment, periodic changes in glycolytic flux and FBP levels induce periodic changes in tissue shape (movies S3 and S4), we found that tissue shape changes are neither necessary nor sufficient for metabolic entrainment of the segmentation clock. Accordingly, we found that periodic pulses of pyruvate caused a similar shape change phenotype without entraining the segmentation clock (movie S5), while periodic pulses of 2,5-AM entrained the segmentation clock without causing tissue shape changes (movie S6).

The precise mechanism by which FBP signals to the segmentation clock network remains to be uncovered. Toward this goal, we studied glycolytic flux-induced transcriptional responses and combined it with a spatially resolved, enhancer-mediated gene-regulatory network (eGRN) analysis in PSM cells. For the eGRN analysis, we used the gene regulatory network inference including enhancers (GRaNIE) method (*31*), which constructs eGRN based on covariation of chromatin [i.e., transcription factor (TF) binding site] accessibility, TF expression, and corresponding target gene expression across samples. The eGRN analysis was done using wild-type PSM tissues that were microdissected into tail bud, posterior PSM, anterior PSM, and somite regions. Using this approach, the resulting eGRN is linked to gene expression changes following PSM cell differentiation along the embryonic axis, which also mirrors metabolic state changes (fig. S4A) (*9*, *20*). The resulting eGRN includes 2522 of the 28,629 (=9%) genes expressed in the PSM and consists of 69 regulons, where each regulon represents a set of target genes regulated by a TF through their accessible enhancer regions. The PSM-specific eGRN was verified using the gene regulatory network performance analysis (GRaNPA) (*31*), which is a machine learning tool that predicts differential expression of genes from another independent experiment and which evaluates the prediction of the network compared to randomly permuted connections. GRaNPA showed that the PSM-specific eGRN that we had identified can predict differentially expressed genes (DEGs) between different regions of the PSM better than a random network (fig. S4B).

The unbiased, paired analysis of these PSM-specific regulons with flux-induced transcriptional changes (table S1) revealed that most of flux-regulated genes are parts of the regulon controlled by the Wnt signaling regulator Tcf7l2 (fig. S5, A, B, and D) (*32*, *33*). The expression of Tcf7l2 regulon was decreased upon glycolytic activation or FBP supplementation (fig. S5E). T cell transcription factors (TCFs) are known to work as transcriptional switches, activating or repressing downstream targets in a context-dependent manner (*34*, *35*). In our eGRN analysis, we identified Tcf7l2 functioning as a repressor (fig. S4A). Intriguingly, we did not identify Tcf7l2 as a flux-responsive gene in our transcriptome dataset (fig. S5C). These results, hence, suggest the presence of a glycolysis-Wnt signaling axis where increased glycolytic flux activates Tcf7l2, which represses the expression of the regulon, providing the potential mechanistic basis for the anticorrelation between glycolytic flux and Wnt signaling target gene expression. Future studies will build on these findings and will be able to address the specific role of Tcf7l2 and the other regulons that we identified in mediating the glycolytic flux/FBP signal.

### Glycolytic flux signaling controls the period of the segmentation clock

We next turned to the functional role of the glycolysis-FBP-Wnt signaling axis and specifically addressed its impact in controlling the period of the segmentation clock. As outlined above, it is critical to tune glycolytic flux within a physiological range and to monitor its impact on the segmentation clock dynamics using a quantitative, real-time imaging approach. To manipulate glycolytic flux in a specific and genetic manner, we used a conditional *cytoPFKFB3* transgenic mouse line (hereafter termed as “TG”) that we generated previously (*11*). In this TG line, a cytoplasmic, dominant-active form of the glycolytic enzyme PFKFB3 (*36*) is expressed from the *Rosa26* locus upon CRE-recombination, leading to a glucose dose–dependent increase of glycolytic flux in PSM explants (*11*).

**Fig. 3.**
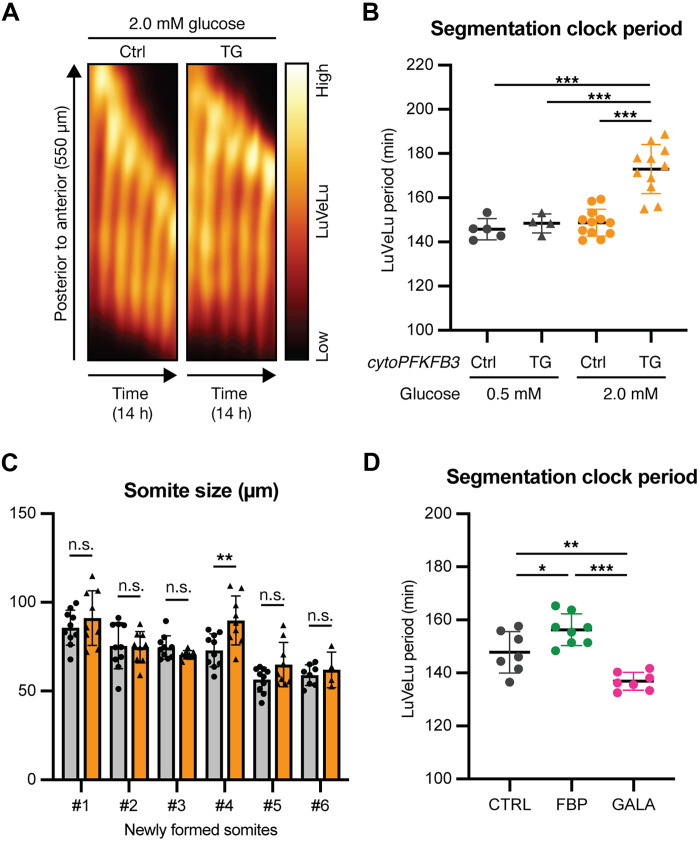
Glycolytic flux tunes the segmentation clock period in an anticorrelated manner. (**A**) Kymographs showing the dynamics of the Notch signaling reporter [=LuVeLu (*65*)] in control (Ctrl) and cytoPFKFB3 (TG) PSM explants in 2.0 mM glucose condition. h, hours. (**B**) Quantification of the segmentation clock period in control (Ctrl) and *cytoPFKFB3* (TG) PSM explants cultured in different glucose conditions. The clock period was determined as a mean of LuVeLu periods between 400 and 600 min of the imaging. Because the clock period is highly sensitive to temperature, the comparisons are always made within each experiment. One-way analysis of variance (ANOVA) with Tukey’s post-hoc test (***P < 0.001). Data are presented as means ± SD, and individual data points represent biological replicates. (**C**) Quantification of somite size in control (Ctrl) and *cytoPFKFB3* (TG) PSM explants cultured in 2.0 mM glucose. The length of newly formed somites along the anteroposterior axis was determined. Somite #1 corresponds to the first somite formed during the time window of live imaging. Welch’s *t*-test (**P < 0.01). Data are presented as means ± SD, and individual data points represent biological replicates. n.s., not significant. (**D**) Quantification of the segmentation clock period in wild-type PSM explants cultured in different metabolic conditions [CTRL, culture medium with 2.0 mM glucose; FBP, culture medium with 2.0 mM glucose and 10 mM FBP; GALA, culture medium with 2.0 mM galactose (without glucose)]. The clock period was determined as a mean of LuVeLu periods between 400 and 600 min of the imaging. One-way ANOVA with Tukey’s post-hoc test (*P < 0.05; **P < 0.01; ***P < 0.001). Data are presented as means ± SD, and individual data points represent biological replicates.

We focused on 2.0 mM glucose condition in which TG explants show an elevated glycolytic flux compared to control samples but show no qualitative difference in PSM patterning (fig. S6A) (*11*). Intriguingly, we found that, in this culture condition, the segmentation clock oscillations were significantly slower (by about 20%) in TG explants, compared to those in control explants (Fig. 3, A and B, and movie S8). This change in oscillation period was accompanied, at morphological level, by a transient increase in the somite length (Fig. 3C). The increase in segmentation clock period was also evident when quantifying Wnt signaling oscillations using a *Axin2-Achilles* knock-in reporter line that we generated previously (fig. S6, B and C, and movie S9) (*19*). To test whether the observed effect on the segmentation clock oscillations is due to an increased glycolytic flux and not merely the effect of the overexpression of cytoPFKFB3 protein per se, we cultured TG explants in reduced glucose concentrations to lower glycolytic flux (fig. S6A). Lowering glucose concentration rescued the clock period phenotype in TG explants (Fig. 3B). This indicated that the segmentation clock period responds to changes in glycolytic flux rather than cytoPFKFB3 protein per se.

We did not find a correlation between cellular redox state and the effect on segmentation clock period. Accordingly, the NAD^+^/NADH ratio was comparable between control and TG explants cultured in 2.0 mM glucose (fig. S6D), although there was a significant difference in the segmentation clock period between these conditions (Fig. 3B). Thus, cellular redox state does not account for glycolytic flux control of the segmentation clock period, which is consistent with our observation that cellular redox changes are not essential for metabolic entrainment of the segmentation clock (fig. S2, A, A′, C, and D).

To test whether glycolytic flux controls the segmentation clock period also in wild-type conditions, we quantified the effect of tuning (i.e., increasing and decreasing) glycolytic flux by titrating glucose concentrations in the culture medium and using real-time imaging of clock oscillations as a quantitative readout. Also in wild-type conditions, we found that the segmentation clock period responded to changes in glycolytic flux. Increasing glucose concentrations led to a slower segmentation clock (fig. S6E). Because we revealed that the glycolytic flux signal is localized at the level of FBP, we directly tested whether supplementation with FBP also affects the clock period. FBP addition led to a slowing down of the segmentation clock (Fig. 3D). We also tested whether a decrease in glycolytic flux would lead to an acceleration of segmentation clock oscillations. To this end, we quantified the oscillation period in samples cultured in galactose-containing medium, because glycolytic flux in this condition is minimal (Fig. 1A). Notably, we found that the segmentation clock oscillated faster in the galactose condition than in the glucose condition (Fig. 3D). Therefore, our results reveal an anticorrelation of glycolytic flux and segmentation clock period: Decreasing glycolytic flux speeds up the segmentation clock and vice versa.

### A genetic rescue experiment demonstrates a noncanonical signaling role of glycolysis

**Fig. 4.**
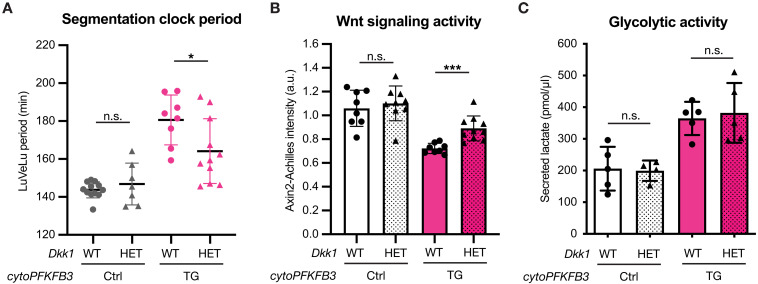
Genetic rescue of the slow segmentation clock phenotype in *cytoPFKFB3* embryos without affecting glycolytic flux. (**A** and **B**) Quantification of the segmentation clock period (A) and Wnt signaling activity (B) in control (Ctrl) and *cytoPFKFB3* (TG) explants with one allele of *Dkk1* (HET), compared to samples with wild-type *Dkk1* copy number (WT). The clock period under 2.0 mM glucose condition was determined as a mean of LuVeLu periods between 400 and 600 min of imaging, while Wnt signaling activity was determined as a mean of the Wnt signaling reporter (=*Axin2-Achilles* knock-in reporter) intensity between 400 and 500 min of imaging. Welch’s unpaired *t*-test (n.s., not significant; *P < 0.05; ***P < 0.001). Data are presented as means ± SD, and individual data points represent biological replicates. a.u., arbitrary unit. (**C**) Lactate secretion was quantified as a proxy for glycolytic flux within PSM cells. After 12 hours of ex vivo culture in 2.0 mM glucose, the amount of lactate secreted from control (Ctrl) and *cytoPFKFB3* (TG) PSM explants was quantified in samples with normal *Dkk1* copy number (WT) and in samples with one allele of *Dkk1* (HET). Welch’s unpaired *t*-test (n.s., not significant). Data are presented as means ± SD, and individual data points represent biological replicates.

To obtain definitive evidence for a signaling role of glycolysis that is mediated via Wnt signaling, we set up a genetic rescue experiment using a mutant for *Dickkopf-1* (*Dkk1*). Dkk1 is a Wnt signaling inhibitor that acts at the level of ligand-receptor interaction and plays a critical role during development (*37*, *38*). Given that increased glycolysis that we found in TG embryos led to decreased Wnt signaling, we asked whether partial deletion of the Wnt signaling inhibitor *Dkk1* could rescue the slow clock phenotype. Excitingly, we found that, in TG embryos in which one allele of *Dkk1* was deleted, not only was Wnt signaling restored, but also the segmentation clock period was rescued in most of the samples (Fig. 4, A and B). Critically, we revealed that *Dkk1* heterozygosity led to a rescue of Wnt signaling and the clock period without affecting glycolytic flux (Fig. 4C). These findings demonstrate that the proximate cause of the observed clock phenotype in TG embryos is a change in Wnt signaling, rather than the cellular bioenergetic/biosynthetic state. In alignment with this, we revealed that phamacological inhibition of Wnt signaling leads to slowing down of the segmentation clock oscillations (fig. S7). We, therefore, conclude that glycolysis exerts a noncanonical signaling role, mediated by the FBP-Wnt signaling axis, regulating the timing of embryo axis segmentation.

## DISCUSSION

### A noncanonical signaling function of glycolytic metabolites during PSM development

In this study, we show that glycolytic flux controls the timing of axis segmentation via a signaling function, which we can decouple from the canonical glycolytic bioenergetic/biosynthetic role. Several findings are of particular importance to substantiate this central conclusion. First, the “subminimal glucose” rescue experiment reveals that, while glycolysis is indispensable, only a minute, energetically subminimal amount of glucose is actually required to support PSM development (Fig. 1, C and D). Second, we showed that periodic supplementation of 2,5-AM, which leads to periodic production of a nonmetabolizable FBP analog, is sufficient to entrain the segmentation clock oscillations (fig. S2, B, B′, and C). Last, we show that the effect of increased glycolytic flux on the timing of the segmentation clock can be rescued by restoring Wnt signaling activity without the need to restore metabolic activity per se (Fig. 4). It is important to stress that, in this genetic rescue condition, only the signaling activity, but not the metabolic state, was restored by *Dkk1* deletion and that such changes in signaling activity were sufficient to rescue the slow clock phenotype. This finding also indicates that glycolytic flux signals, at least, in part, through the Wnt signaling pathway. Although numbers are small, we noticed a potential bimodal rescue response in *Dkk1* heterozygous *cytoPFKFB3* embryos, the underlying cause remains to be investigated.

Previously, several mechanisms have been proposed regarding how glucose metabolism affects signaling via its effect on posttranslational modifications, including acetylation of ß-catenin (*10*, *39*, *40*). However, in the experimental context of mouse embryo PSM that we study, we did not find a clear correlation between glycolytic flux and acetylation of ß-catenin (fig. S3). Instead, our results presented here reveal a noncanonical signaling role for glycolysis that is centered around the metabolite FBP. Key findings supporting this conclusion are the effect of FBP supplementation on the clock period (Fig. 3D) and the potent entrainment of the segmentation clock by periodic pulses of FBP (Fig. 2, C and C′). Our finding that the glycolytic flux signaling function centers on the metabolite FBP is also particularly interesting given that FBP serves as a sentinel metabolite from bacteria to mammals (*11*, *27*). Accordingly, the FBP-Wnt signaling axis, functionally integrates the status of glycolytic flux, while its signaling mechanism can be decoupled from the bioenergetic and biosynthetic roles of glycolysis.

The detailed molecular mechanisms by which intracellular FBP levels are sensed and linked to downstream responses is yet unknown and a key question that needs to be addressed in future studies. The physiological concentrations of FBP in developing mouse embryos remain unknown and, hence, need to be determined in the future studies. Moreover, it will be critical to probe allosteric metabolite (FBP)–protein interactions, as FBP has already been shown to have widespread potential to allosterically affect protein function (*41*–*44*). We speculate that one protein controlled by FBP could be Tcf7l2, as our transcriptome and gene regulatory network analyses identified targets of Tcf7l2 as particularly glycolytic flux sensitive (fig. S5). Notably, Tcf7l2 has been strongly associated with type 2 diabetes and is involved in glucose homeostasis and insulin secretion in pancreatic β cells (*45*, *46*). It will, hence, be of particular importance to test a potential FBP-Tcf7l2 interaction not just in the context of the segmentation clock network but also potentially in other biological contexts, including pancreatic β cells.

### Identification of a functional anticorrelation between glycolytic flux/FBP and Wnt signaling

Combined with our previous study (*11*), we provide evidence that glycolysis controls Wnt signaling in a dose-dependent and anticorrelated manner (Fig. 4B and fig. S5). Hence, while increasing glycolytic flux/FBP leads to a decrease in Wnt signaling target gene expression and a slowing down of segmentation tempo, we also see evidence for the inverse: Decreasing glycolytic flux within a physiological range correlates with increased Wnt target gene expression and accelerated segmentation. Furthermore, we showed that periodic application of FBP synchronizes first Wnt signaling oscillations, while Notch signaling oscillations become entrained with a delay (Fig. 2, C and D). We interpret these findings as an indication of the specificity of the FBP signal toward the Wnt signaling pathway. This FBP-Wnt signaling axis controls the timing of embryo axis segmentation. While a previous study reported that, in chick embryo PSM, glycolysis functions downstream of the FGF signaling pathway (*20*), this functional dependency awaits testing in the mouse PSM explant model. Along this line, further studies are also needed to understand how FBP signaling modulates the shape of Wnt signaling gradient, reflecting graded expression of Wnt ligands within the PSM.

Our results from studying PSM explant samples appear to contrast with findings from several studies that used in vitro stem cell models for mesoderm specification. In several reports, glycolytic inhibition led to down-regulation, not up-regulation, of Wnt signaling (*10*, *20*, *47*–*49*). One potential reason for this apparent discrepancy could be rooted in the differences in the experimental systems. While all samples are cultured in vitro, in our study, we used primary PSM explants that are cultured in a chemically defined medium without exogenous signaling or growth factors, and, hence, all signals are originating from the embryonic cells and tissue (we, hence, suggest to term these endogenous in vitro assays). In contrast, stem cell and organoid cultures rely on a successful combination of a series of external growth and signaling factors and, thus, many experimental parameters, including their concentrations, timing of addition, and duration of treatment, have critical impacts on the phenotypic outcome. We suggest to term these assays requiring external control exogenous in vitro assays. Another source potentially explaining the observed discrepancy might be found at the level of the strength and specificity (and lack thereof) of metabolic perturbations used in the above studies. In the studies mentioned above, glycolysis was mainly impaired pharmacologically by using 2-deoxyglucose, which is known to have pleiotropic effects on metabolism (*50*). This metabolic impairment was found to be correlated with a down-regulation of cellular signaling activity, including, but not limited to, Wnt signaling. In contrast, when we tune glycolytic flux, using glucose titration for instance, we find an anticorrelated response at the level of Wnt signaling targets and segmentation clock period (figs. S5 and S6E) (*11*). Therefore, in future studies, it will be critical to apply metabolic perturbations with high specificity and to remain within a physiological range, as demonstrated in this study, to be able to compare outcomes across different contexts.

### Glycolysis-FBP-Wnt signaling axis controls the segmentation clock period

The primary function of the glycolysis-FBP-Wnt signaling axis that we revealed in this study is the control of the segmentation clock period in mouse embryos (Figs. 3 and 4). Intriguingly, in *cytoPFKFB3* explants with higher glycolytic flux, a slowing down of the segmentation clock led to an increase in somite size but only for the fourth somites that they formed during the ex vivo culture (Fig. 3C). This observation is not in agreement with the classical clock and wavefront model supported by previous studies (*16*, *51*–*53*), which predicts a stable change in somite size upon a stable change in the clock period. Further investigations based on the entrainment approach presented here are needed to reveal the underlying mechanisms. Previously, Wnt signaling had been functionally linked to the regulation of the segmentation clock period (*54*), although the underlying mechanisms were not addressed. Consistent with this, we revealed the functional link between Wnt signaling and the segmentation clock rhythm (fig. S7). Moreover, our work reveals the direct impact of the metabolic state on Wnt signaling and clock period, emphasizing the need for future studies to identify how Wnt signaling affects the period of segmentation clock oscillations.

Recently, a series of studies have reported potential mechanisms of how the oscillation period can be tuned experimentally. Accordingly, a study using exogenous in vitro stem cell system reported that the segmentation clock period can be controlled by mitochondrial respiration, cellular redox state, and, ultimately, protein translation rate (*21*). Additionally, several exogenous in vitro studies emphasized that differences in protein turnover rates underlie species-specific developmental timing (*21*, *55*–*58*). Our results revealing a noncanonical signaling function of glycolysis argue for a distinct mechanism of how the clock period is controlled in the mouse embryo PSM. First, as summarized above, our combined results demonstrate that the clock tuning function by glycolysis/FBP can be clearly decoupled from its bioenergetic and biosynthetic role (Figs. 1 and 4). In addition, we were able to localize the signal to the level of FBP and the committed part of glycolysis. Our observation that the fructose analog 2,5-AM, which is converted into nonmetabolizable FBP, can efficiently entrain the segmentation clock strongly supports this argument (fig. S2, B, B′, and C). In contrast, periodic pulses of pyruvate were not sufficient to entrain the segmentation clock, despite its impact on cellular redox state (fig. S2, A, A′, and C). Combined, our findings, hence, argue against a signaling function directly linked to cellular redox state in the control of clock period in the mouse embryo PSM system that we studied. Rather, we demonstrate that a glycolysis-FBP-Wnt signaling axis controls the timing of PSM segmentation.

### Future direction

The renewed interest in the role of metabolism is providing clear evidence for its central role(s) in signaling, cell differentiation, development, and disease (*59*). However, given the multitude and interconnected functions of metabolism, the main challenge and question in the field remains how and whether these distinct functions (e.g., bioenergetic/biosynthetic and signaling functions) can be decoupled. Here, we demonstrate that this challenge can be tackled by using specific metabolic perturbations, tailored genetic models, and metabolic entrainment. This allowed us to reveal that metabolism shows a modular functional organization. We found a noncanonical signaling module in glycolysis controlling the timing of axis segmentation, and this module can be dissociated from bioenergetic/biosynthetic metabolic functions. This approach can now be adapted to other biological contexts to investigate how widespread metabolic modules are in place and how canonical and noncanonical signaling modules are interconnected.

Moreover, our findings raise a more general question regarding the function of the metabolic signaling module per se. One appealing hypothesis is that the intrinsic temporal organization of metabolism, which manifests as metabolic rhythms at various temporal scales (*60*), serves as the core template and signal for biological timing and rhythms (*61*). In this regard, our first demonstration of metabolic entrainment of the segmentation clock provides concrete support for this idea, i.e., ultradian and/or circadian metabolic rhythms could function as intrinsic zeitgeber entraining segmentation clock oscillations. Previous work has already indicated that metabolism is tightly regulated in time and also space in developmental systems (*3*, *9*, *20*, *62*). As a next important step, efforts within the field need to be intensified to test for the presence of spatiotemporal metabolic rhythms in developmental systems.

In addition, the metabolic signaling module could also enable organisms to integrate external environmental cues, such as nutritional resources. This capacity to link milieu extérieur and milieu intérieur (*63*) via a metabolic signal could be relevant to the optimization of life history traits, such as the rate of developmental transitions and body size. Future comparative studies, hence, need to explore the function of noncanonical metabolite signaling during the dynamic interplay of organisms with their natural environment considering the entire life cycle.

## MATERIALS AND METHODS

### Animal work

All animals were housed in the European Molecular Biology Laboratory (EMBL) animal facility under veterinarians’ supervision and were treated following the guidelines of the European Commission, revised directive 2010/63/EU, and American Veterinary Medical Association guidelines 2007. All the animal experiments were approved by the EMBL Institutional Animal Care and Use Committee (project code: 21–001_HD_AA). The detection of a vaginal plug was designated as embryonic day 0.5 (E0.5), and all experiments were conducted with E10.5 embryos.

### Mouse lines

The following mice used in this study were described previously and were genotyped using primers described in these references: *Axin2-Achilles* (*19*), *HprtCre* (*64*), *LuVeLu* (*65*), *Rosa26-loxP-stop-loxP-cytoPFKFB3* (*11*), and *Dkk1* mutant (*38*). While the *Dkk1* mutant line was maintained on C57BL/6j genetic background, the other mouse lines were maintained on CD1 genetic background. For the genetic rescue experiments, the following primers were used to detect the mutant allele of *Dkk1* (*66*): forward, 5′-GCT CTA ATG CTC TAG TGC TCT AGT GAC- 3′; and reverse, 5′-GTA GAA TTG ACC TGC AGG GGC CCT CGA-3′.

### Ex vivo culture of PSM explants

Dissection and ex vivo culture of PSM explants were performed as described before (*11*). In brief, E10.5 embryos were collected in Dulbecco’s modified Eagle’s medium (DMEM)/F12 (without glucose, pyruvate, glutamine, and phenol red; Cell Culture Technologies) supplemented with 2.0 mM glucose (Sigma-Aldrich, G8769), 2.0 mM glutamine (Sigma-Aldrich, G7513), 1.0% (w/v) bovine serum albumin (BSA; Cohn fraction V; Equitech-Bio, BAC62), and 10 mM Hepes (Gibco, 15360–106). PSM explants were isolated using a micro scalpel (Feather Safety Razor, No. 715, 02.003.00.715) and were cultured in DMEM/F12 supplemented with 0.5 to 2.0 mM glucose, 2.0 mM glutamine, and 1.0% (w/v) BSA (Cohn fraction V; Equitech-Bio, BAC62) at 37°C, under 5% CO_2_ and 60% O_2_ condition. For Wnt inhibition, IWP-2 (Sigma-Aldrich, I0536) was added to the culture medium. For the rescue experiments with a subminimal amount of glucose, PSM explants were collected in DMEM/F12 supplemented with 0.01% (w/v) BSA and 10 mM Hepes (without glucose, pyruvate, and glutamine) and were cultured in DMEM/F12 supplemented with 0.025 mM glucose and/or 20 mM pyruvate (without glutamine and BSA).

### Live imaging of Notch and Wnt signaling reporter lines

To monitor Notch and Wnt signaling activity using real-time imaging, *LuVeLu* (*65*) and *Axin2-Achilles* knock-in (*19*) reporter lines were used, respectively. Following dissection, PSM explants were washed once with culture medium and were transferred into agar wells (600-nm width, 3% low melting point agarose; Biozyme, 840101) in four-well slides (Lab-Tek, no. 155383). Imaging was performed with a LSM780 laser scanning microscope (Zeiss), at 37°C, under 5% CO_2_ and 65% O_2_ condition. Samples were excited by a 514-nm wavelength argon laser through 20× Plan-Apochromat objective (numerical aperture, 0.8). Image processing was done using the Fiji software (*67*). For extracting the period and phase of signaling oscillations, wavelet analysis was performed using pyBOAT (*68*).

For the quantification of Notch and Wnt signaling oscillations, kymographs were first generated from time-series images that were registered using the MultiStackReg plugin and Gaussian blurred. Reporter intensities in the posterior (for Notch oscillations) and the anterior (for Wnt oscillations) PSM were extracted along lines drawn in parallel to growing posterior ends of the tissues or the regressing determination front, respectively. The intensity profiles were analyzed using a wavelet analysis workflow (*68*) to extract periods of Notch and Wnt signaling oscillations. For the comparison of Wnt signaling activity, moving regions of interest (ROIs; 30 pixels in diameter) were placed in the posterior PSM to obtain intensity profiles of the *Axin2-Achilles* knock-in reporter over time. For each experiment, the intensity values were normalized to the value from wild-type embryos at the first time point, and the datasets from different experiments were combined for statistical analysis.

### Metabolic measurements

PSM explants without somites were cultured in DMEM/F12 supplemented with varying amounts of glucose or galactose (Sigma-Aldrich, G0750). The explants were flash frozen by liquid N_2_ following 1 hour of ex vivo culture and were stored at −80°C until use. NAD^+^/NADH measurements were performed according to the manufacturer’s instructions (Promega, G9071). In brief, eight explants were lysed in 90 μl of 0.1 M NaOH with 0.5% DTAB and were split into two tubes (40 μl per tube). Samples were then incubated at 60°C for 15 min with or without adding 20 μl of 0.4 M HCl for NAD^+^ and NADH measurements, respectively. After neutralization by either 0.5 M Trizma base solution (for NAD^+^ samples) or Trizma-HCl solution (for NADH samples), the lysates were used for NAD^+^/NADH measurements. For ATP/ADP measurements (Abcam, ab65313), the explants were dissociated immediately following 1 hour ex vivo culture in cold phosphate-buffered saline (PBS) containing 0.01% BSA (two explants in 4 μl of PBS) and were processed for the analysis according to the manufacturer’s instructions. Lactate measurements were performed as described before (*11*).

### ATAC- and RNA-sequencing analysis

PSM explants of E10.5 wild-type embryos (CD1 genetic background) were microdissected into tail bud, posterior PSM, anterior PSM, and somite regions by micro scalpel in cold PBS. Each tissue region was transferred into a micro well (ibidi, no. 80486) and mechanically dissociated to a cell suspension in 4.2 μl cold PBS. Last, 0.7 μl and 3.3 μl cell suspensions were used for RNA sequencing (RNA-seq) and assay of transposase accessible chromatin (ATAC) sequencing (ATAC-seq), respectively. For the comparison between control and *cytoPFKFB3* PSM explants, explants were cultured for 3 hours ex vivo before collecting tail buds for RNA-seq analysis.

#### ATAC sequencing

We followed the Omni-ATAC protocol (*69*) with some modifications. For transposition reactions, 3.3 μl of cell suspensions were mixed with 5.0 μl of 2× TD buffer [20 mM Tris-HCl (pH 7.6), 10 mM MgCl_2_, and 20% dimethyl formamide], 1.0 μl of TDE1 (Illumina, no. 15027865), 0.1 μl of 1% digitonin (Promega, no. G9441), 0.1 μl of 10% Tween 20 (Sigma-Aldrich, no. 11332465001), 0.1 μl of 10% NP-40 (Sigma-Aldrich, no. 11332473001), and 0.4 μl of nuclease-free water. After 30 min of incubation at 37°C on a thermomixer set at 600 rpm, the samples were purified by a DNA Clean and Concentrator-5 (Zymo Research, D4014), and DNA concentrations were determined by the Qubit Fluorometer (dsDNA High Sensitivity Kit, Thermo Fisher Scientific, Q32851). The samples were diluted to 20 ng/μl and used as templates for library preparations by polymerase chain reaction (PCR). PCR reactions were performed using primers from the Nextera XT Index Kit (Illumina, FC-131-1001) and NEBNext High Fidelity 2X PCR Master Mix (New England Biolabs, M0541). After purification with the QIAGEN MinElute PCR Purification Kit (QIAGEN, 28004), individual libraries were size selected [100 to 800 base pairs (bp)] with AMPure XP beads (Beckman Coulter, no. A63881). Libraries were quantified using the Qubit Fluorometer (dsDNA High Sensitivity Kit) and average fragment length distribution was determined by the Bioanalyzer (Agilent, High Sensitivity DNA kit, 5067-4626). Prepared libraries were multiplexed in pools of equimolar concentrations and sequenced on the NextSeq 500 (Illumina) platform with 75-bp paired-end readings. After demultiplexing and barcode trimming (Trimmomatic Galaxy version 0.36.6), sequencing reads were quality checked (FastQC Galaxy Version 0.73) and aligned to *Mus musculus* genome (GRCm38) with the Bowtie2 aligner (Galaxy version 2.3.4.2, options -I 0 -X 2000 –dovetail –sensitive). Multimapping and duplicate reads were removed, and, last, only reads mapping to major chromosomes were kept (*70*).

#### RNA sequencing

We followed the Smart-seq2 protocol (*71*) with some modifications. In brief, dissociated cells were lysed with three times the volume of cell lysis buffer (0.02% Triton X-100 with RNasin), snap frozen by liquid N_2_, and stored at −80°C until cDNA synthesis. cDNAs were synthesized using SuperScript IV Reverse Transcriptase (Thermo Fisher Scientific) and amplified by PCR with HiFi Kapa Hot start ReadyMix (Kapa Biosystems, KK2601). After cleanup with SPRI beads, concentrations of cDNA (50 to 9000 bp) samples were determined by the Bioanalyzer (Agilent, High Sensitivity DNA kit). cDNAs (250 pg) were then used for tagmentation-based library preparation. Libraries were quantified using the Qubit Fluorometer (dsDNA High Sensitivity Kit), and average fragment length distribution was determined by the Bioanalyzer (Agilent, High Sensitivity DNA kit, 5067-4626). Prepared libraries were multiplexed in pools of equimolar concentrations and sequenced on the NextSeq 500 (Illumina) with 75-bp paired-end (for the wild-type, noncultured PSM explants) or single-end (for the comparison between control and *cytoPFKFB3* explants) readings. After demultiplexing and barcode trimming (TrimGalore Galaxy version 0.4.3.1), sequencing reads were quality checked (FastQC Galaxy version 0.69) and aligned to *M. musculus* genome (GRCm38) with the STAR aligner (version 2.5.2b, default options) (*70*). Multimapping reads were removed, and RNA-seq quality was assessed with Picard CollectRnaSeqMetrics (Galaxy version 2.7.1.1)

### GRaNIE and GRaNPA analysis

eGRN was constructed from the matched RNA-seq and ATAC-seq data (24 samples for each) of the PSM explants from E10.5 wild-type embryos using the developer’s version of the now published GRaNIE package (https://bioconductor.org/packages/release/bioc/html/GRaNIE.html) (*31*). Raw gene counts from RNA-seq data were produced with a summarizeOverlaps function from the GenomicAlignments R package (https://bioconductor.org/packages/release/bioc/html/GenomicAlignments.html) (*72*), corrected for different experimental batches using Combat-seq function from the R package sva (*73*), and log_2_ normalized. ATAC-seq peak counts were generated using DiffBind R package (https://bioconductor.org/packages/DiffBind/), and peak positions were identified using MACS2 software (https://genomebiology.biomedcentral.com/articles/10.1186/gb-2008-9-9-r137) (*74*). The details of the GRaNIE approach are described here (*31*). Briefly, in the first step, the expression of each TF was correlated with the accessibility of each of the accessible regions (=ATAC-seq peak) with and without a known binding site of the TF (foreground and background, respectively). Known binding sites were defined using the HOCOMOCO database v.10 (*75*). Significantly correlated TF-peak links were identified using empirical false discovery rate of 30% (calculated separately for each TF) and an absolute correlation Pearson’s coefficient of >0.4. In the second step, chromatin accessibility at the ATAC-seq peaks was correlated with the expression of all genes less than 250 kb away from the peak and peak-gene links were retained if they were positively and significantly (*P* < 0.05) correlated (our assumption is that accessibility at the regulatory region positively correlates with expression of the linked gene) and if their Pearson’s correlation coefficient was >0.4. This resulted in the eGRN consisting of 69 TFs, 5154 TF-peak-gene connections of 2522 unique genes. TF regulons were defined as all TF-gene links of each TF within the network. We performed the GRaNPA (https://git.embl.de/grp-zaugg/GRaNPA) to evaluate the biological relevance of our eGRN. To this end, we used the published dataset from Chal *et al.* (*76*) and selected DEGs (381 genes in total) from their list of DEGs between the anterior and the posterior PSM by filtering them with *P* value of <0.05 and adjusted *P* value of <0.2.

### Microfluidics-based segmentation clock entrainment

Polydimethylsiloxane (PDMS) chips and polytetrafluoroethylene (PTFE) tubing (inner diameter, 0.6 mm; APT AWG24T) for microfluidics-based entrainment experiments were prepared as described before (*14*, *19*). Culture media were prepared on the day of experiments by adding a metabolite of interest [either glucose, FBP (Santa Cruz Biotechnology, sc-221476), pyruvate (Sigma-Aldrich, P4562), 2,5-AM (Cayman, 21673), or 3-OMG (Sigma-Aldrich, M4879)] to DMEM/F12 supplemented with 2.0 mM glutamine (Sigma-Aldrich, G7513), 0.01% (w/v) BSA (Cohn fraction V; Equitech-Bio, BAC62), and 1% penicillin-streptomycin (Gibco, 15140122). The PDMS chip (soaked in PBS) and the culture medium (filled in 10-mL syringes; BD Biosciences, 300912; diameter, 14.5 mm) were degassed before use for at least 1 hour in a vacuum desiccator chamber.

Following dissection, PSM explants with two intact somites were transferred to the PDMS chip, and sample inlets were plugged with a PDMS-filled PTFE tubing. The tubings connected to the syringes with medium were then connected to the medium inlets, and the samples were placed in the incubator (37°C, 5% CO_2_, and 65% O_2_) installed on a LSM780 laser scanning microscope (Zeiss) for preculture. Pumping was started for both the control and treatment medium at the flow rate of 20 μl/hour. A half-hour later, only the control medium was pumped into the chip for another 30 min at the flow rate of 60 μl/hour. After the preculture, imaging was started under constant or alternating culture conditions.

For data analysis, moving ROIs (30 pixels in diameter) were placed in the posterior PSM to obtain intensity profiles of LuVeLu or Axin2-Achilles reporters over time. To extract the period and phase of LuVeLu and Axin2-Achilles oscillations, the intensity profiles were analyzed using a wavelet analysis workflow (*68*). Entrainment of Notch and Wnt signaling oscillations was analyzed using stroboscopic maps and the first Kuramoto order parameter as described before (*19*).

### Western blot analysis

Tail bud regions were collected and snap frozen by liquid N_2_ for Western blot analysis following 3-hour ex vivo culture in desired culture conditions. For the preparation of lysates, two tail buds were lysed in 10 μl of radioimmunoprecipitation assay (RIPA) buffer containing a cOmplete protease inhibitor cocktail (Merck, 11873580001). Primary antibodies used in the study are as follows: anti–ß-catenin antibody (Thermo Fisher Scientific, 13-8400; RRID:AB_2533039, 1:1000), anti–acetyl-ß-catenin antibody (Cell Signaling Technology, 9534; RRID:AB_823679; 1:1000), and anti–ß-tubulin (Millipore, 05-661; RRID:AB_309885; 1:2000).

## References

[1] M. G. Vander Heiden, L. C. Cantley, C. B. Thompson, Understanding the Warburg effect: The metabolic requirements of cell proliferation. Science 324, 1029–1033 (2009). 19460998 10.1126/science.1160809 PMC2849637

[2] M. A. Reid, Z. Dai, J. W. Locasale, The impact of cellular metabolism on chromatin dynamics and epigenetics. Nat. Cell Biol. 19, 1298–1306 (2017). 29058720 10.1038/ncb3629 PMC5886854

[3] H. Miyazawa, A. Aulehla, Revisiting the role of metabolism during development. Development 145, dev131110 (2018). 30275240 10.1242/dev.131110

[4] A. M. Intlekofer, L. W. S. Finley, Metabolic signatures of cancer cells and stem cells. Nat. Metab. 1, 177–188 (2019). 31245788 10.1038/s42255-019-0032-0 PMC6594714

[5] X. Yang, M. Heinemann, J. Howard, G. Huber, S. Iyer-Biswas, G. L. Treut, M. Lynch, K. L. Montooth, D. J. Needleman, S. Pigolotti, J. Rodenfels, P. Ronceray, S. Shankar, I. Tavassoly, S. Thutupalli, D. V. Titov, J. Wang, P. J. Foster, Physical bioenergetics: Energy fluxes, budgets, and constraints in cells. Proc. Natl. Acad. Sci. U.S.A. 118, e2026786118 (2021). 34140336 10.1073/pnas.2026786118 PMC8255778

[6] C. Pan, B. Li, M. C. Simon, Moonlighting functions of metabolic enzymes and metabolites in cancer. Mol. Cell 81, 3760–3774 (2021). 34547237 10.1016/j.molcel.2021.08.031

[7] S. A. Baker, J. Rutter, Metabolites as signalling molecules. Nat. Rev. Mol. Cell Biol. 24, 355–374 (2023). 36635456 10.1038/s41580-022-00572-w

[8] P. Liberali, A. F. Schier, The evolution of developmental biology through conceptual and technological revolutions. Cell 187, 3461–3495 (2024). 38906136 10.1016/j.cell.2024.05.053

[9] V. Bulusu, N. Prior, M. T. Snaebjornsson, A. Kuehne, K. F. Sonnen, J. Kress, F. Stein, C. Schultz, U. Sauer, A. Aulehla, Spatiotemporal analysis of a glycolytic activity gradient linked to mouse embryo mesoderm development. Dev. Cell 40, 331–341.e4 (2017). 28245920 10.1016/j.devcel.2017.01.015 PMC5337618

[10] M. Oginuma, Y. Harima, O. A. Tarazona, M. Diaz-Cuadros, A. Michaut, T. Ishitani, F. Xiong, O. Pourquié, Intracellular pH controls Wnt downstream of glycolysis in amniote embryos. Nature 584, 98–101 (2020). 32581357 10.1038/s41586-020-2428-0 PMC8278564

[11] H. Miyazawa, M. T. Snaebjornsson, N. Prior, E. Kafkia, H. M. Hammarén, N. Tsuchida-Straeten, K. R. Patil, M. Beck, A. Aulehla, Glycolytic flux-signaling controls mouse embryo mesoderm development. eLife 11, e83299 (2022). 36469462 10.7554/eLife.83299 PMC9771359

[12] O. F. Venzin, A. C. Oates, What are you synching about? Emerging complexity of Notch signaling in the segmentation clock. Dev. Biol. 460, 40–54 (2020). 31302101 10.1016/j.ydbio.2019.06.024

[13] A. Hubaud, O. Pourquie, Signalling dynamics in vertebrate segmentation. Nat. Rev. Mol. Cell Biol. 15, 709–721 (2014). 25335437 10.1038/nrm3891

[14] K. F. Sonnen, V. M. Lauschke, J. Uraji, H. J. Falk, Y. Petersen, M. C. Funk, M. Beaupeux, P. François, C. A. Merten, A. Aulehla, Modulation of phase shift between Wnt and Notch signaling oscillations controls mesoderm segmentation. Cell 172, 1079–1090.e12 (2018). 29474908 10.1016/j.cell.2018.01.026 PMC5847172

[15] M. F. Simsek, A. S. Chandel, D. Saparov, O. Q. H. Zinani, N. Clason, E. M. Özbudak, Periodic inhibition of Erk activity drives sequential somite segmentation. Nature 613, 153–159 (2023). 36517597 10.1038/s41586-022-05527-x PMC9846577

[16] J. Cooke, E. C. Zeeman, A clock and wavefront model for control of the number of repeated structures during animal morphogenesis. J. Theor. Biol. 58, 455–476 (1976). 940335 10.1016/s0022-5193(76)80131-2

[17] Y. Niwa, H. Shimojo, A. Isomura, A. González, H. Miyachi, R. Kageyama, Different types of oscillations in Notch and Fgf signaling regulate the spatiotemporal periodicity of somitogenesis. Genes Dev. 25, 1115–1120 (2011). 21632822 10.1101/gad.2035311 PMC3110950

[18] A. Aulehla, C. Wehrle, B. Brand-Saberi, R. Kemler, A. Gossler, B. Kanzler, B. G. Herrmann, Wnt3a plays a major role in the segmentation clock controlling somitogenesis. Dev. Cell 4, 395–406 (2003). 12636920 10.1016/s1534-5807(03)00055-8

[19] P. G. L. Sanchez, V. Mochulska, C. Mauffette Denis, G. Mönke, T. Tomita, N. Tsuchida-Straeten, Y. Petersen, K. Sonnen, P. François, A. Aulehla, Arnold tongue entrainment reveals dynamical principles of the embryonic segmentation clock. eLife 11, e79575 (2022). 36223168 10.7554/eLife.79575 PMC9560162

[20] M. Oginuma, P. Moncuquet, F. Xiong, E. Karoly, J. Chal, K. Guevorkian, O. Pourquié, A gradient of glycolytic activity coordinates FGF and Wnt signaling during elongation of the body axis in amniote embryos. Dev. Cell 40, 342–353.e10 (2017). 28245921 10.1016/j.devcel.2017.02.001 PMC5403012

[21] M. Diaz-Cuadros, T. P. Miettinen, O. S. Skinner, D. Sheedy, C. M. Díaz-García, S. Gapon, A. Hubaud, G. Yellen, S. R. Manalis, W. M. Oldham, O. Pourquié, Metabolic regulation of species-specific developmental rates. Nature 613, 550–557 (2023). 36599986 10.1038/s41586-022-05574-4 PMC9944513

[22] M. B. Renfree, H. C. Hensleigh, A. McLaren, Developmental changes in the composition and amount of mouse fetal fluids. J. Embryol. Exp. Morphol. 33, 435–446 (1975). 51898

[23] D. A. New, Development of explanted rat embryos in circulating medium. J. Embryol. Exp. Morphol. 17, 513–525 (1967). 4860575

[24] P. P. Tam, The control of somitogenesis in mouse embryos. J. Embryol. Exp. Morphol. 65, 103–128 (1981). 6801176

[25] R. Rossignol, R. Gilkerson, R. Aggeler, K. Yamagata, S. J. Remington, R. A. Capaldi, Energy substrate modulates mitochondrial structure and oxidative capacity in cancer cells. Cancer Res. 64, 985–993 (2004). 14871829 10.1158/0008-5472.can-03-1101

[26] N. T. Spratt Jr., Nutritional requirement of the early chick embryo. III. the metabolic basis of the morphogenesis and differentiation as revealed by the use of inhibitors. Biol. Bull. 99, 120–135 (1950). 14772248 10.2307/1538756

[27] K. Kochanowski, B. Volkmer, L. Gerosa, B. R. Haverkorn van Rijsewijk, A. Schmidt, M. Heinemann, Functioning of a metabolic flux sensor in *Escherichia coli*. Proc. Natl. Acad. Sci. U.S.A. 110, 1130–1135 (2013). 23277571 10.1073/pnas.1202582110 PMC3549114

[28] P. T. Riquelme, M. E. Wernette-Hammond, N. M. Kneer, H. A. Lardy, Regulation of carbohydrate metabolism by 2,5-anhydro-D-mannitol. Proc. Natl. Acad. Sci. U.S.A. 80, 4301–4305 (1983). 6410389 10.1073/pnas.80.14.4301 PMC384025

[29] P. T. Riquelme, M. E. Wernette-Hammond, N. M. Kneer, H. A. Lardy, Mechanism of action of 2,5-anhydro-D-mannitol in hepatocytes. effects of phosphorylated metabolites on enzymes of carbohydrate metabolism. J. Biol. Chem. 259, 5115–5123 (1984). 6325420

[30] P. T. Riquelme, N. M. Kneer, M. E. Wernette-Hammond, H. A. Lardy, Inhibition by 2,5-anhydromannitol of glycolysis in isolated rat hepatocytes and in Ehrlich ascites cells. Proc. Natl. Acad. Sci. U.S.A. 82, 78–82 (1985). 3155858 10.1073/pnas.82.1.78 PMC396974

[31] A. Kamal, C. Arnold, A. Claringbould, R. Moussa, N. H. Servaas, M. Kholmatov, N. Daga, D. Nogina, S. Mueller-Dott, A. Reyes-Palomares, G. Palla, O. Sigalova, D. Bunina, C. Pabst, J. B. Zaugg, GRaNIE and GRaNPA: Inference and evaluation of enhancer-mediated gene regulatory networks. Mol. Syst. Biol. 19, e11627 (2023). 37073532 10.15252/msb.202311627 PMC10258561

[32] H. Clevers, R. Nusse, Wnt/β-catenin signaling and disease. Cell 149, 1192–1205 (2012). 22682243 10.1016/j.cell.2012.05.012

[33] M. L. Angus-Hill, K. M. Elbert, J. Hidalgo, M. R. Capecchi, T-cell factor 4 functions as a tumor suppressor whose disruption modulates colon cell proliferation and tumorigenesis. Proc. Natl. Acad. Sci. U.S.A. 108, 4914–4919 (2011). 21383188 10.1073/pnas.1102300108 PMC3064334

[34] K. M. Cadigan, TCFs and Wnt/β-catenin signaling: More than one way to throw the switch. Curr. Top. Dev. Biol. 98, 1–34 (2012). 22305157 10.1016/B978-0-12-386499-4.00001-X

[35] A.-B. Ramakrishnan, A. Sinha, V. B. Fan, K. M. Cadigan, The Wnt transcriptional switch: TLE removal or inactivation? Bioessays 40, 10.1002/bies.201700162 (2018). 10.1002/bies.201700162 PMC7495026 29250807

[36] A. Yalcin, B. F. Clem, A. Simmons, A. Lane, K. Nelson, A. L. Clem, E. Brock, D. Siow, B. Wattenberg, S. Telang, J. Chesney, Nuclear targeting of 6-phosphofructo-2-kinase (PFKFB3) increases proliferation via cyclin-dependent kinases. J. Biol. Chem. 284, 24223–24232 (2009). 19473963 10.1074/jbc.M109.016816 PMC2782016

[37] C. Niehrs, Function and biological roles of the Dickkopf family of Wnt modulators. Oncogene 25, 7469–7481 (2006). 17143291 10.1038/sj.onc.1210054

[38] M. Mukhopadhyay, S. Shtrom, C. Rodriguez-Esteban, L. Chen, T. Tsukui, L. Gomer, D. W. Dorward, A. Glinka, A. Grinberg, S. P. Huang, C. Niehrs, J. C. I. Belmonte, H. Westphal, Dickkopf1 is required for embryonic head induction and limb morphogenesis in the mouse. Dev. Cell 1, 423–434 (2001). 11702953 10.1016/s1534-5807(01)00041-7

[39] A. Chocarro-Calvo, J. M. Garcia-Martinez, S. Ardila-Gonzalez, A. De la Vieja, C. Garcia-Jimenez, Glucose-induced β-catenin acetylation enhances Wnt signaling in cancer. Mol. Cell 49, 474–486 (2013). 23273980 10.1016/j.molcel.2012.11.022

[40] S. H. Anagnostou, P. R. Shepherd, Glucose induces an autocrine activation of the Wnt/β-catenin pathway in macrophage cell lines. Biochem. J. 416, 211–218 (2008). 18823284 10.1042/BJ20081426

[41] I. Piazza, K. Kochanowski, V. Cappelletti, T. Fuhrer, E. Noor, U. Sauer, P. Picotti, A map of protein-metabolite interactions reveals principles of chemical communication. Cell 172, 358–372.e23 (2018). 29307493 10.1016/j.cell.2017.12.006

[42] K. G. Hicks, A. A. Cluntun, H. L. Schubert, S. R. Hackett, J. A. Berg, P. G. Leonard, M. A. Ajalla Aleixo, Y. Zhou, A. J. Bott, S. R. Salvatore, F. Chang, A. Blevins, P. Barta, S. Tilley, A. Leifer, A. Guzman, A. Arok, S. Fogarty, J. M. Winter, H. C. Ahn, K. N. Allen, S. Block, I. A. Cardoso, J. Ding, I. Dreveny, W. C. Gasper, Q. Ho, A. Matsuura, M. J. Palladino, S. Prajapati, P. Sun, K. Tittmann, D. R. Tolan, J. Unterlass, A. P. VanDemark, M. G. Vander Heiden, B. A. Webb, C. H. Yun, P. Zhao, B. Wang, F. J. Schopfer, C. P. Hill, M. C. Nonato, F. L. Muller, J. E. Cox, J. Rutter, Protein-metabolite interactomics of carbohydrate metabolism reveal regulation of lactate dehydrogenase. Science 379, 996–1003 (2023). 36893255 10.1126/science.abm3452 PMC10262665

[43] K. Peeters, F. van Leemputte, B. Fischer, B. M. Bonini, H. Quezada, M. Tsytlonok, D. Haesen, W. Vanthienen, N. Bernardes, C. B. Gonzalez-Blas, V. Janssens, P. Tompa, W. Versées, J. M. Thevelein, Fructose-1,6-bisphosphate couples glycolytic flux to activation of ras. Nat. Commun. 8, 922 (2017). 29030545 10.1038/s41467-017-01019-z PMC5640605

[44] W.-J. Zhou, H.-L. Yang, J. Mei, K.-K. Chang, H. Lu, Z.-Z. Lai, J.-W. Shi, X.-H. Wang, K. Wu, T. Zhang, J. Wang, J.-S. Sun, J.-F. Ye, D.-J. Li, J.-Y. Zhao, L.-P. Jin, M.-Q. Li, Fructose-1,6-bisphosphate prevents pregnancy loss by inducing decidual COX-2^+^ macrophage differentiation. Sci. Adv. 8, eabj2488 (2022). 35196096 10.1126/sciadv.abj2488 PMC8865779

[45] G. da Silva Xavier, M. K. Loder, A. McDonald, A. I. Tarasov, R. Carzaniga, K. Kronenberger, S. Barg, G. A. Rutter, TCF7l2 regulates late events in insulin secretion from pancreatic islet β-cells. Diabetes 58, 894–905 (2009). 19168596 10.2337/db08-1187 PMC2661588

[46] L. Del Bosque-Plata, E. Martinez-Martinez, M. A. Espinoza-Camacho, C. Gragnoli, The role of *TCF7l2* in type 2 diabetes. Diabetes 70, 1220–1228 (2021). 34016596 10.2337/db20-0573 PMC8275893

[47] K. S. Stapornwongkul, E. Hahn, P. Poliński, L. Salamó Palau, K. Arató, L. A. Yao, K. Williamson, N. Gritti, K. Anlas, M. Osuna Lopez, K. R. Patil, I. Heemskerk, M. Ebisuya, V. Trivedi, Glycolytic activity instructs germ layer proportions through regulation of nodal and Wnt signaling. Cell Stem Cell 32, 744–758.e7 (2025). 40245870 10.1016/j.stem.2025.03.011 PMC12048219

[48] A. Villaronga-Luque, R. G. Savill, N. López-Anguita, A. Bolondi, S. Garai, S. I. Gassaloglu, R. Rouatbi, K. Schmeisser, A. Poddar, L. Bauer, T. Alves, S. Traikov, J. Rodenfels, T. Chavakis, A. Bulut-Karslioglu, J. V. Veenvliet, Integrated molecular-phenotypic profiling reveals metabolic control of morphological variation in a stem-cell-based embryo model. Cell Stem Cell 32, 759–777.e13 (2025). 40245869 10.1016/j.stem.2025.03.012

[49] C. Dingare, J. Yang, B. Steventon, Mannose is crucial for mesoderm specification and symmetry breaking in gastruloids. bioRxiv 543730 [Preprint] (2023). 10.1101/2023.06.05.543730. PMC7619011 38636516

[50] C. Laussel, S. Leon, Cellular toxicity of the metabolic inhibitor 2-deoxyglucose and associated resistance mechanisms. Biochem. Pharmacol. 182, 114213 (2020). 32890467 10.1016/j.bcp.2020.114213

[51] C. Schroter, A. C. Oates, Segment number and axial identity in a segmentation clock period mutant. Curr. Biol. 20, 1254–1258 (2010). 20637625 10.1016/j.cub.2010.05.071

[52] L. Herrgen, S. Ares, L. G. Morelli, C. Schröter, F. Jülicher, A. C. Oates, Intercellular coupling regulates the period of the segmentation clock. Curr. Biol. 20, 1244–1253 (2010). 20637620 10.1016/j.cub.2010.06.034

[53] Y. Harima, Y. Takashima, Y. Ueda, T. Ohtsuka, R. Kageyama, Accelerating the tempo of the segmentation clock by reducing the number of introns in the *Hes7* gene. Cell Rep. 3, 1–7 (2013). 23219549 10.1016/j.celrep.2012.11.012

[54] S. Gibb, A. Zagorska, K. Melton, G. Tenin, I. Vacca, P. Trainor, M. Maroto, J. K. Dale, Interfering with Wnt signalling alters the periodicity of the segmentation clock. Dev. Biol. 330, 21–31 (2009). 19272372 10.1016/j.ydbio.2009.02.035 PMC2686089

[55] T. Rayon, D. Stamataki, R. Perez-Carrasco, L. Garcia-Perez, C. Barrington, M. Melchionda, K. Exelby, J. Lazaro, V. L. J. Tybulewicz, E. M. C. Fisher, J. Briscoe, Species-specific pace of development is associated with differences in protein stability. Science 369, eaba7667 (2020). 32943498 10.1126/science.aba7667 PMC7116327

[56] M. Matsuda, H. Hayashi, J. Garcia-Ojalvo, K. Yoshioka-Kobayashi, R. Kageyama, Y. Yamanaka, M. Ikeya, J. Toguchida, C. Alev, M. Ebisuya, Species-specific segmentation clock periods are due to differential biochemical reaction speeds. Science 369, 1450–1455 (2020). 32943519 10.1126/science.aba7668

[57] R. Iwata, P. Casimir, E. Erkol, L. Boubakar, M. Planque, I. M. Gallego López, M. Ditkowska, V. Gaspariunaite, S. Beckers, D. Remans, K. Vints, A. Vandekeere, S. Poovathingal, M. Bird, I. Vlaeminck, E. Creemers, K. Wierda, N. Corthout, P. Vermeersch, S. Carpentier, K. Davie, M. Mazzone, N. V. Gounko, S. Aerts, B. Ghesquière, S. M. Fendt, P. Vanderhaeghen, Mitochondria metabolism sets the species-specific tempo of neuronal development. Science 379, eabn4705 (2023). 36705539 10.1126/science.abn4705

[58] J. Lázaro, M. Costanzo, M. Sanaki-Matsumiya, C. Girardot, M. Hayashi, K. Hayashi, S. Diecke, T. B. Hildebrandt, G. Lazzari, J. Wu, S. Petkov, R. Behr, V. Trivedi, M. Matsuda, M. Ebisuya, A stem cell zoo uncovers intracellular scaling of developmental tempo across mammals. Cell Stem Cell 30, 938–949.e7 (2023). 37343565 10.1016/j.stem.2023.05.014 PMC10321541

[59] A. M. Garfinkel, E. Ilker, H. Miyazawa, K. Schmeisser, J. M. Tennessen, Historic obstacles and emerging opportunities in the field of developmental metabolism – Lessons from Heidelberg. Development 151, dev202937 (2024). 38912552 10.1242/dev.202937 PMC11299503

[60] A. Goldbeter, M. J. Berridge, *Biochemical Oscillations and Cellular Rhythms: The Molecular Bases of Periodic and Chaotic Behaviour* (Cambridge Univ. Press, 1996).

[61] B. P. Tu, S. L. McKnight, Metabolic cycles as an underlying basis of biological oscillations. Nat. Rev. Mol. Cell Biol. 7, 696–701 (2006). 16823381 10.1038/nrm1980

[62] D. Cao, J. Bergmann, L. Zhong, A. Hemalatha, C. Dingare, T. Jensen, A. L. Cox, V. Greco, B. Steventon, B. Sozen, Selective utilization of glucose metabolism guides mammalian gastrulation. Nature 634, 919–928 (2024). 39415005 10.1038/s41586-024-08044-1 PMC11499262

[63] C. Bernard, *Introduction à l’Étude de la Médecine Expérimentale* (Le Livre de Poche, 2011).

[64] S. H. Tang, F. J. Silva, W. M. Tsark, J. R. Mann, A cre/loxp-deleter transgenic line in mouse strain 129s1/svimj. Genesis 32, 199–202 (2002). 11892008 10.1002/gene.10030

[65] A. Aulehla, W. Wiegraebe, V. Baubet, M. B. Wahl, C. Deng, M. Taketo, M. Lewandoski, O. Pourquié, A β-catenin gradient links the clock and wavefront systems in mouse embryo segmentation. Nat. Cell Biol. 10, 186–193 (2008). 18157121 10.1038/ncb1679 PMC7391962

[66] M. D. Phillips, M. Mukhopadhyay, C. Poscablo, H. Westphal, Dkk1 and dkk2 regulate epicardial specification during mouse heart development. Int. J. Cardiol. 150, 186–192 (2011). 20439124 10.1016/j.ijcard.2010.04.007 PMC2916964

[67] J. Schindelin, I. Arganda-Carreras, E. Frise, V. Kaynig, M. Longair, T. Pietzsch, S. Preibisch, C. Rueden, S. Saalfeld, B. Schmid, J. Y. Tinevez, D. J. White, V. Hartenstein, K. Eliceiri, P. Tomancak, A. Cardona, Fiji: An open-source platform for biological-image analysis. Nat. Methods 9, 676–682 (2012). 22743772 10.1038/nmeth.2019 PMC3855844

[68] G. Mönke, F. Sorgenfrei, C. Schmal, A. Granada, Optimal time frequency analysis for biological data - pyBOAT. bioRxiv 067744 [Preprint] (2020). 10.1101/2020.04.29.067744.

[69] M. R. Corces, A. E. Trevino, E. G. Hamilton, P. G. Greenside, N. A. Sinnott-Armstrong, S. Vesuna, A. T. Satpathy, A. J. Rubin, K. S. Montine, B. Wu, A. Kathiria, S. W. Cho, M. R. Mumbach, A. C. Carter, M. Kasowski, L. A. Orloff, V. I. Risca, A. Kundaje, P. A. Khavari, T. J. Montine, W. J. Greenleaf, H. Y. Chang, An improved ATAC-seq protocol reduces background and enables interrogation of frozen tissues. Nat. Methods 14, 959–962 (2017). 28846090 10.1038/nmeth.4396 PMC5623106

[70] A. Dobin, C. A. Davis, F. Schlesinger, J. Drenkow, C. Zaleski, S. Jha, P. Batut, M. Chaisson, T. R. Gingeras, Star: Ultrafast universal RNA-seq aligner. Bioinformatics 29, 15–21 (2013). 23104886 10.1093/bioinformatics/bts635 PMC3530905

[71] S. Picelli, O. R. Faridani, Å. K. Björklund, G. Winberg, S. Sagasser, R. Sandberg, Full-length RNA-seq from single cells using Smart-seq2. Nat. Protoc. 9, 171–181 (2014). 24385147 10.1038/nprot.2014.006

[72] M. Lawrence, W. Huber, H. Pagès, P. Aboyoun, M. Carlson, R. Gentleman, M. T. Morgan, V. J. Carey, Software for computing and annotating genomic ranges. PLOS Comput. Biol. 9, e1003118 (2013). 23950696 10.1371/journal.pcbi.1003118 PMC3738458

[73] J. T. Leek, J. D. Storey, Capturing heterogeneity in gene expression studies by surrogate variable analysis. PLOS Genet. 3, 1724–1735 (2007). 17907809 10.1371/journal.pgen.0030161 PMC1994707

[74] Y. Zhang, T. Liu, C. A. Meyer, J. Eeckhoute, D. S. Johnson, B. E. Bernstein, C. Nusbaum, R. M. Myers, M. Brown, W. Li, X. S. Liu, Model-based analysis of ChIP-Seq (MACS). Genome Biol. 9, R137 (2008). 18798982 10.1186/gb-2008-9-9-r137 PMC2592715

[75] I. V. Kulakovskiy, I. E. Vorontsov, I. S. Yevshin, R. N. Sharipov, A. D. Fedorova, E. I. Rumynskiy, Y. A. Medvedeva, A. Magana-Mora, V. B. Bajic, D. A. Papatsenko, F. A. Kolpakov, V. J. Makeev, Hocomoco: Towards a complete collection of transcription factor binding models for human and mouse via large-scale ChIP-Seq analysis. Nucleic Acids Res. 46, D252–D259 (2018). 29140464 10.1093/nar/gkx1106 PMC5753240

[76] J. Chal, M. Oginuma, Z. al Tanoury, B. Gobert, O. Sumara, A. Hick, F. Bousson, Y. Zidouni, C. Mursch, P. Moncuquet, O. Tassy, S. Vincent, A. Miyanari, A. Bera, J. M. Garnier, G. Guevara, M. Hestin, L. Kennedy, S. Hayashi, B. Drayton, T. Cherrier, B. Gayraud-Morel, E. Gussoni, F. Relaix, S. Tajbakhsh, O. Pourquié, Differentiation of pluripotent stem cells to muscle fiber to model duchenne muscular dystrophy. Nat. Biotechnol. 33, 962–969 (2015). 26237517 10.1038/nbt.3297

