# Supplementary material for: A noncanonical role of glycolytic metabolites controlling the timing of mouse embryo segmentation
Source: Sci Adv. 2025 Sep 19;11(38):eadz9606. doi: 10.1126/sciadv.adz9606 (PMC12448123; doi:10.1126/sciadv.adz9606)
Supplement: Supplementary file 1 — Figs. S1 to S7 Legend for table S1 Legends for movies S1 to S9 [file sciadv.adz9606_sm.pdf]

Science Advances
AAAS

Supplementary Materials for

A noncanonical role of glycolytic metabolites controlling the timing of mouse embryo segmentation

Hidenobu Miyazawa *et al.*

Corresponding author: Hidenobu Miyazawa, hidenobu.miyazawa@embl.de; Alexander Aulehla, aulehla@embl.de

Sci. Adv. **11**, eadz9606 (2025)
DOI: 10.1126/sciadv.adz9606

The PDF file includes:

Figs. S1 to S7
Legend for table S1
Legends for movies S1 to S9

Other Supplementary Material for this manuscript includes the following:

Table S1
Movies S1 to S9

Figure S1

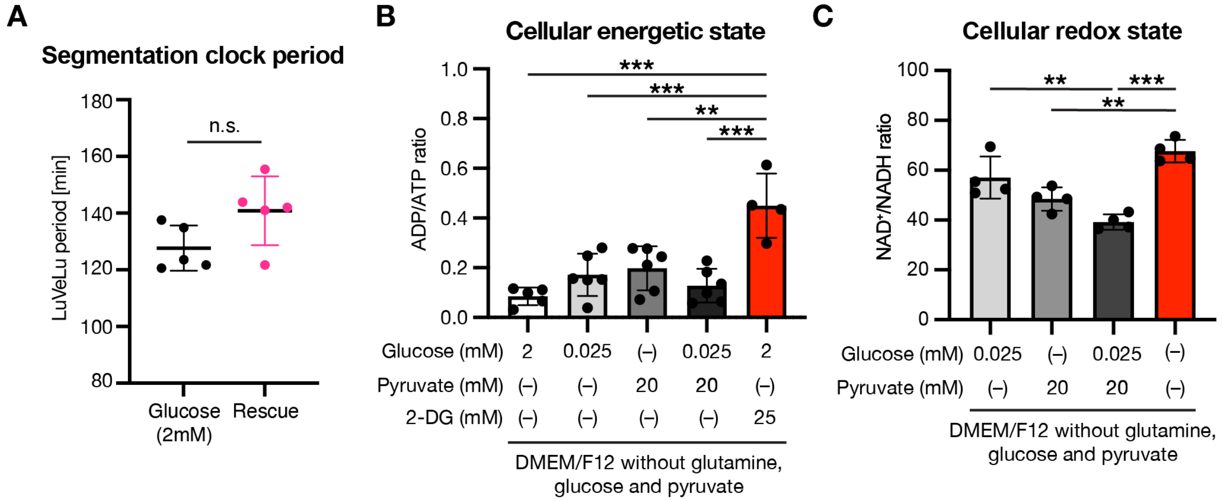

Fig. S1. The effects of a sub-minimal amount of glucose on the segmentation clock oscillations and bioenergetic state of PSM cells. (A) Quantification of the segmentation clock period in control (2.0 mM glucose) and the rescue (0.025 mM glucose plus 20 mM pyruvate) conditions. The clock period was determined as a mean of LuVeLu periods between 400-600 min of the imaging. Unpaired Welch's t-test (n.s., not significant). Data are presented as mean ± standard deviation (SD), and individual data points represent biological replicates. **(B,C)** The effect of a subminimal amount of glucose on cellular bioenergetic state. ADP/ATP (B) and NAD^+/NADH (C) ratios were measured using whole tissue lysates following one-hour ex vivo culture of wild-type PSM explants in various culture conditions. Data are presented as mean ± SD, and individual data points represent biological replicates. One-way ANOVA with Tukey's post hoc test, **p <0.01, ***p <0.001.

Figure S2

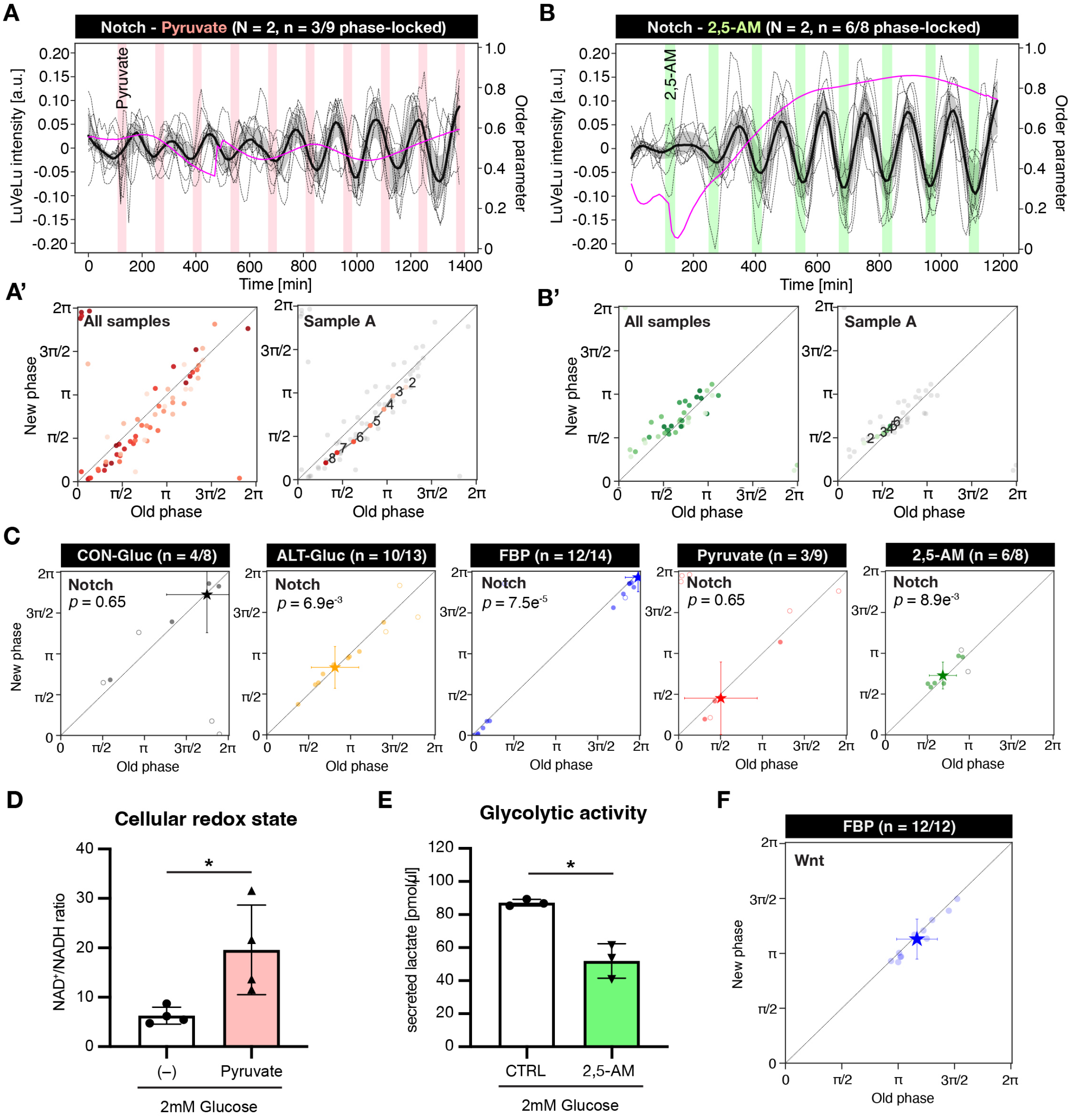

Fig. S2. Segmentation clock entrainment by periodic, transient glycolytic cues. (A,B) Detrended (via sinc-filter detrending, cut-off period = 240 min) time-series of LuVeLu intensity oscillations in wild-type PSM explants exposed to periodic pulses of 20 mM pyruvate (A) or

20 mM 2,5-AM (B) (dashed lines: individual samples, bold black line: median values, grey shades: the first to third quartile range). Changes in the first Kuramoto order parameter are shown in magenta. To keep the molarity of the medium constant during experiments, 20 mM non-metabolizable glucose (i.e., 3-O-methyl-D-glucopyranose) was added to the basal medium containing 2.0 mM glucose. **(A',B')** Stroboscopic maps showing step-wise changes in the phase of LuVeLu oscillations in response to periodic pyruvate pulses. Darker dots represent later time points (the numbers in the plots indicate the number of the pulses). **(C)** Stroboscopic maps showing the phase of Notch (i.e., LuVeLu) oscillations at the last pulse of metabolite. Filled circles represent entrained samples, while open circles represent non-entrained samples. Samples are considered to be entrained when a phase difference between the last and second last pulses is less than $\pi/8$. In addition, the Rayleigh test for uniformity was used to statistically evaluate whether entrained samples show non-uniform phase distribution for the last two pulses. CON- Gluc, constant (2.0 mM) glucose condition; ALT-Gluc, alternating (from 2.0 mM to 0.5 mM) glucose condition. **(D)** Quantification of NAD^+/NADH ratio following one-hour ex vivo culture of wild-type PSM explants in the presence or the absence of pyruvate. 20 mM of pyruvate was supplemented to the culture medium containing 2.0 mM glucose. Whole tissue lysates were used for NAD^+/NADH measurements. Data are presented as mean ± standard deviation (SD), and individual data points represent biological replicates. Welch's unpaired t-test, *$p < 0.05$.

(E) The effect of 2,5-AM on glycolytic flux in wild-type PSM explants. The amount of lactate secreted from PSM explants during a 3 h ex vivo culture was quantified as a proxy for glycolytic flux. 20 mM of 2,5-AM was supplemented to DMEM/F12 containing 2.0 mM glucose. Welch's unpaired t-test, *$p < 0.05$. Data are presented as mean ± SD, and individual data points represent biological replicates. **(F)** Stroboscopic maps showing the phase of Wnt (i.e., Axin2-Achilles) oscillations at the last FBP pulse. Filled circles represent entrained samples, while open circles represent non-entrained samples. Samples are considered to be entrained when a

phase difference between the last and second last pulses is less then $\pi/8$.

Figure S3

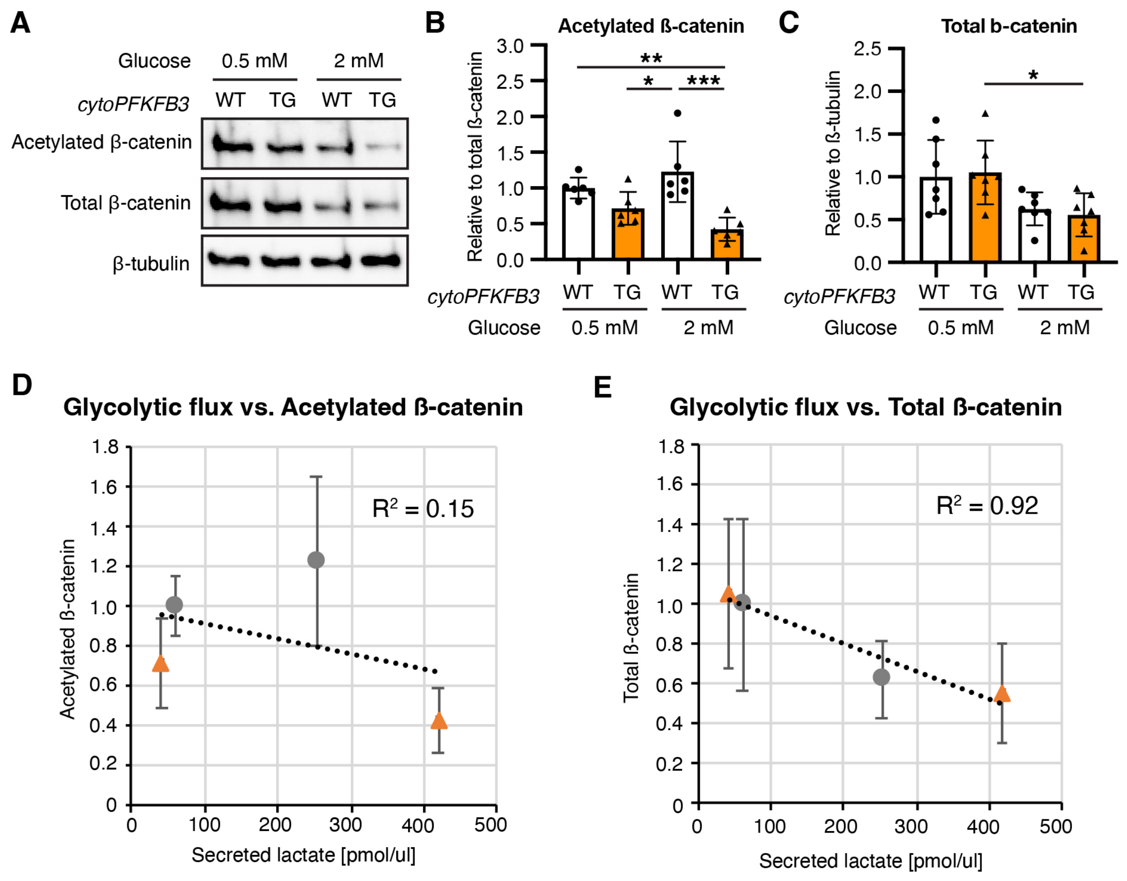

Fig. S3. The effect of changing glycolytic flux on acetylation of ß-catenin in wild-type and cytoPFKFB3 PSM explants. (A–C) Western blot analysis of total and acetylated ß-catenin in wild-type (WT) and cytoPFKFB3 (TG) PSM explants cultured in 0.5 mM or 2 mM glucose for three hours. The relative amounts of acetylated (B) and total (C) ß-catenin were quantified. Data are presented as mean ± standard deviation (SD), and individual data points represent biological replicates. One-way ANOVA with Tukey's post hoc test, *$p < 0.05$, **$p < 0.01$, ***$p < 0.001$. **(D, E)** Correlations between glycolytic flux and acetylated (D) or total (E) ß-catenin levels. The data of secreted lactate is from fig. S6A. Means of the relative acetylated/total ß-catenin are plotted as a function of means of lactate secretion. Linear regression lines were fitted to the data

(Circles: WT; Triangles: TG).

Figure S4

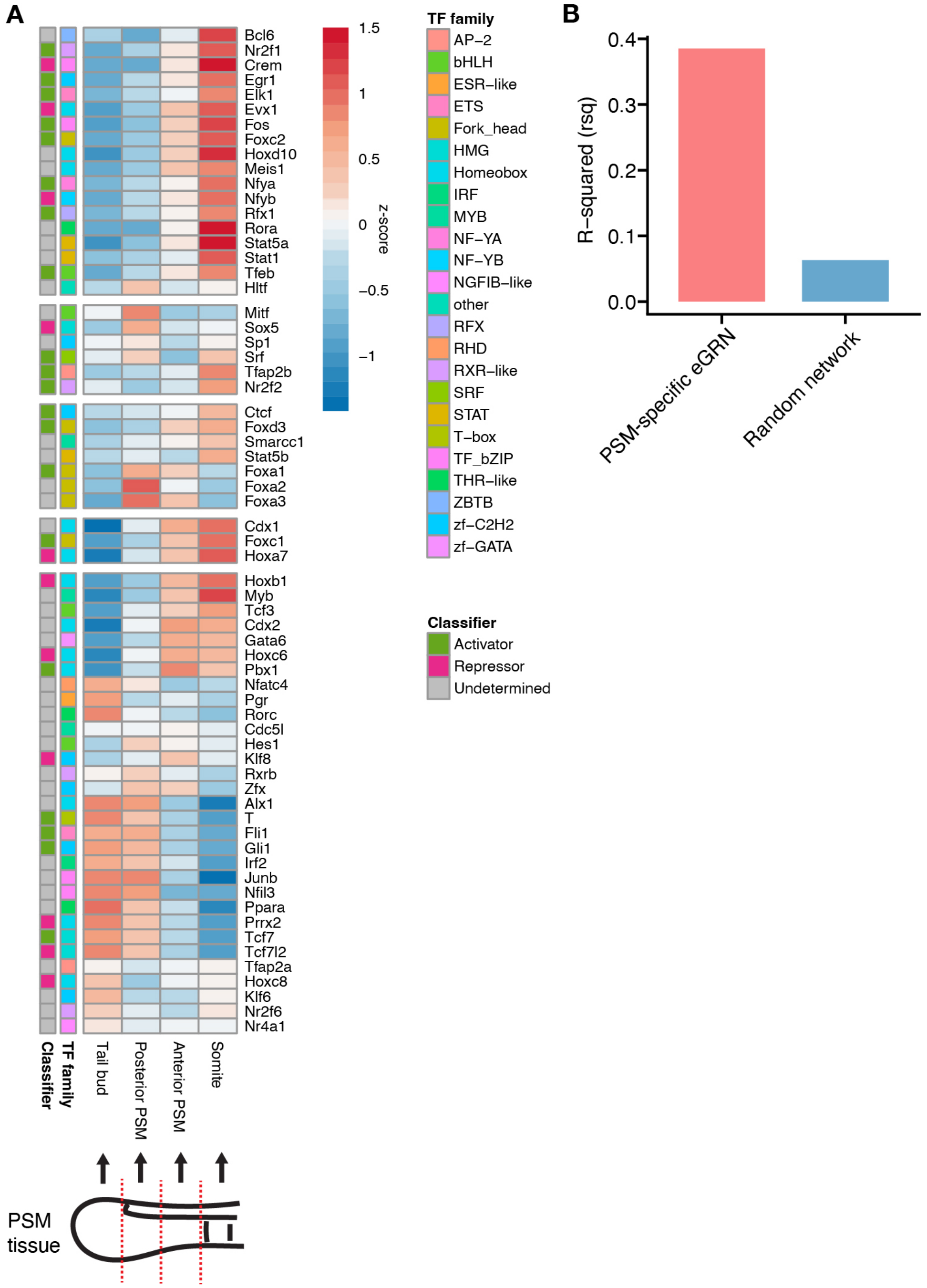

Fig. S4. Building a PSM-specific eGRN using the GRaNIE method. (A) A heatmap showing gene expressions of each PSM-specific regulon (i.e., means of all the targets) identified by the

GRaNIE method. Normalized counts by variance stabilizing transformation (VST) were used to calculate the z-scores. **(B)** GRaNPA evaluation of the PSM-specific eGRNs using the published transcriptome dataset from the microdissected mouse PSM tissues (*76*). Differentially expressed genes between the anterior and posterior PSM (381 DEGs in total) were used to compare the prediction performance between the PSM-specific eGRNs and a random network. R^2 values were calculated based on the plot showing true versus predicted log2 fold changes for DEGs.

Figure S5

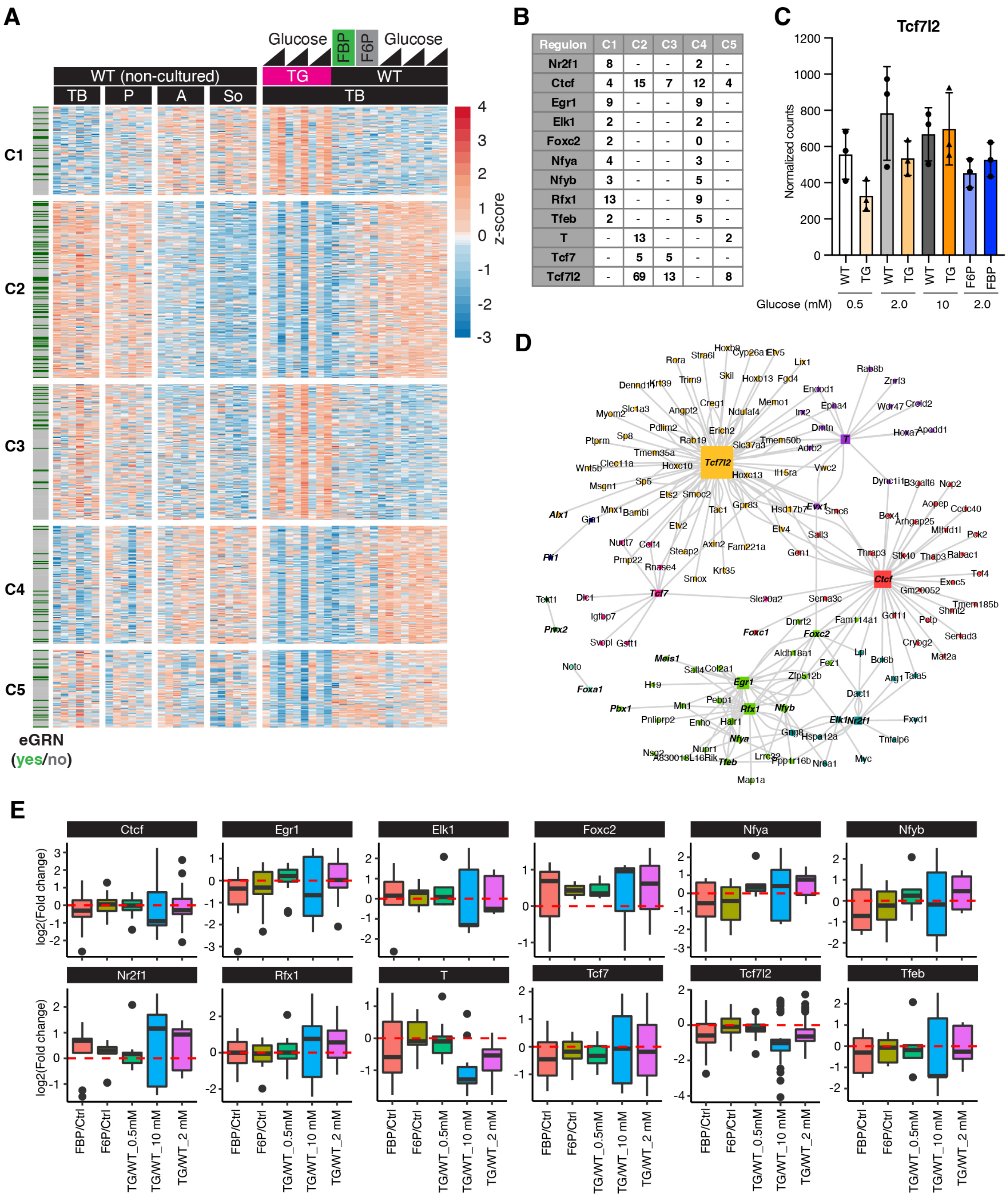

Fig. S5. Tcf7l2 regulon responds to glycolytic flux changes within PSM cells. (A) A

heatmap showing glycolytic flux-responsive differentially expressed genes (DEGs) between wild-type (WT) and cytoPFKFB3 (TG) PSM explants cultured for three-hour in various (i.e., 0.5 mM, 2.0 mM, and 10 mM) glucose conditions (adjusted *p*-value <0.01, WT vs. TG for each glucose condition). Normalized counts by variance stabilizing transformation (VST) were used to calculate the z-scores. The datasets were integrated with the datasets from Miyazawa *et al.* (2022) (*11*). DEGs that are parts of the PSM-specific eGRN are marked by green. **(B)** A table showing the number of the flux-responsive DEGs that are included in each PSM-specific regulon. **(C)** The expression levels of Tcf7l2 in wild-type (WT) and cytoPFKFB3 (TG) explants cultured in different metabolic conditions. Normalized counts are shown (F6P, wild-type explants cultured with 20 mM F6P; FBP, wild-type tailbud cultured with 20 mM FBP). **(D)** A network showing TFs (colored squares) and their glycolytic flux-responsive target genes (colored circles). **(E)** Box plots showing fold changes in gene expressions of flux-sensitive DEGs that constitute each PSM-specific regulon. The fold changes were calculated between different metabolic conditions.

Figure S6

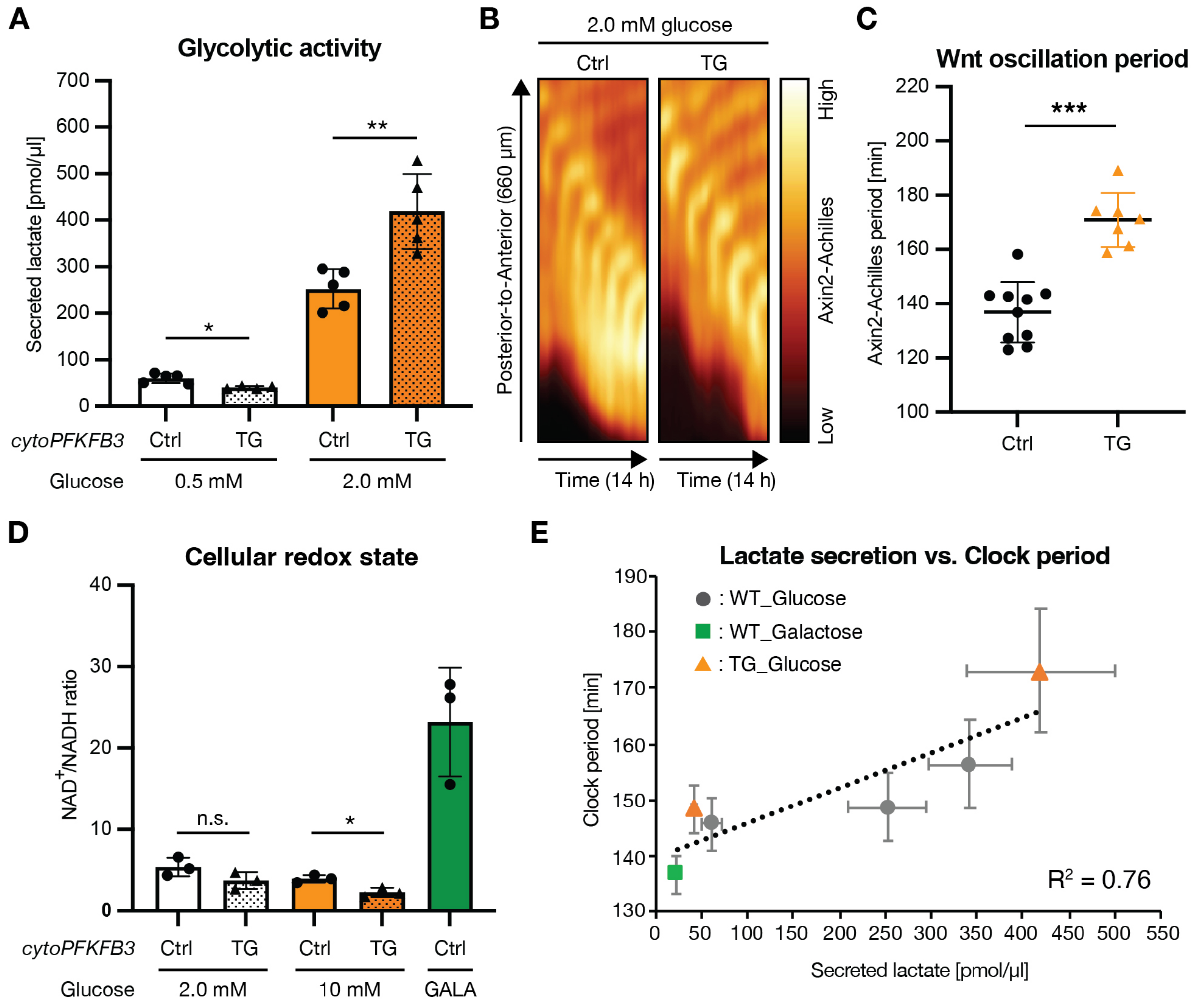

Fig. S6. The effects of tuning glycolytic flux on the segmentation clock oscillations and cellular redox state of PSM explants. (A) Comparison of lactate secretion between control (Ctrl) and cytoPFKFB3 (TG) explants cultured in 0.5 mM or 2.0 mM glucose (the data for 2.0 mM glucose condition is adapted from Miyazawa et al. 2022 (*11*)). Lactate secretion was quantified as a proxy for glycolytic flux within PSM cells. The amount of lactate secreted from PSM explants during 12 h ex vivo culture was quantified. Unpaired Welch's t-test (n.s., not significant). Data are presented as mean ± standard deviation (SD), and individual data points

represent biological replicates. **(B)** Kymographs showing the dynamics of the Axin2-Achilles knock-in reporter in control (Ctrl) and cytoPFKFB3 (TG) PSM explants in 2.0 mM glucose condition. **(C)** Quantifying the period of Wnt signaling oscillations in Ctrl and TG explants cultured in 2.0 mM glucose. The period was determined as a mean of Axin2-Achilles periods between 400-600 min of the imaging. Welch's unpaired t-test, ***$p < 0.001$. Data are presented as mean ± SD, and individual data points represent biological replicates. **(D)** Quantification of NAD^+/NADH ratio following one-hour ex vivo culture of control (Ctrl) and cytoPFKFB3 (TG) PSM explants under various culture conditions. For the galactose (GALA) condition, 2.0 mM galactose was supplemented to the culture medium instead of glucose. Whole tissue lysates were used for NAD^+/NADH measurements. Data are presented as mean ± SD, and individual data points represent biological replicates. Welch's unpaired t-test, *$p < 0.05$. **(E)** A correlation between glycolytic flux and the segmentation clock period. Means of the segmentation clock periods are plotted as a function of means of lactate secretion. A linear regression line was fitted to the data points. WT_Glucose, wild-type explants cultured with either 0.5 mM or 2.0 mM or 25 mM glucose; WT_Galactose, wild-type explants cultured with 2.0 mM galactose; TG_Glucose, cytoPFKFB3 explants cultured with either 0.5 mM or 2.0 mM glucose. The lactate secretion data for WT_Glucose and TG_Glucose was adapted from our previous paper (*11*).

Figure S7

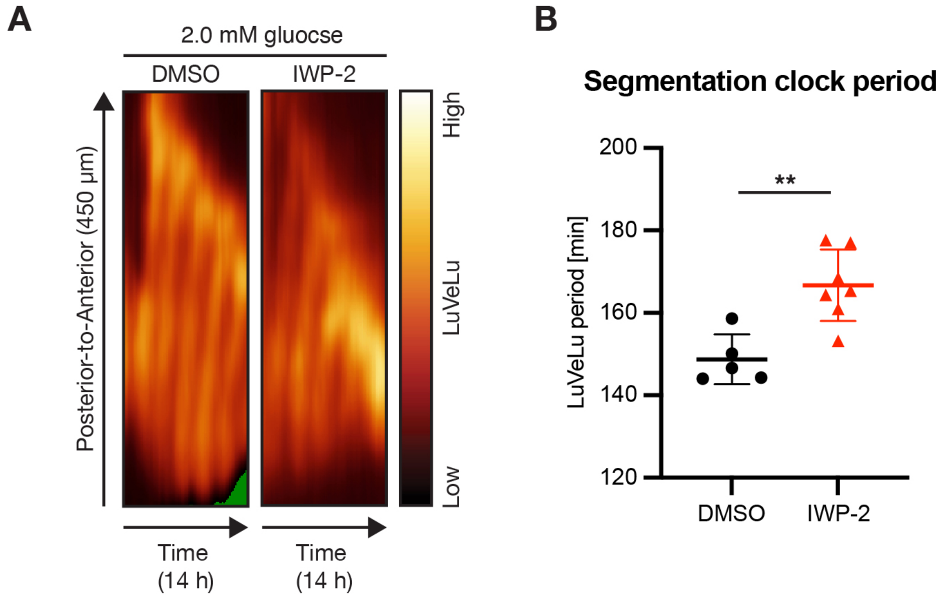

Fig. S7. Wnt inhibition causes slowing down of the segmentation clock oscillations. (A) Kymographs showing the dynamics of the Notch signaling reporter (= LuVeLu) in PSM explants treated with or without a Wnt signaling inhibitor IWP-2 (2 µM). **(B)** Quantification of the segmentation clock period in PSM explants treated with or without IWP-2. Welch's unpaired t-test (**p <0.01). Data are presented as mean ± standard deviation (SD), and individual data points represent biological replicates.

Table S1. Glycolytic flux-responsive genes in the PSM cells. A list of differentially expressed genes between wild-type (WT) and cytoPFKFB3 (TG) PSM explants cultured for three-hour in various (i.e., 0.5 mM, 2.0 mM, and 10 mM) glucose conditions (adjusted p-value <0.01, WT vs. TG for each glucose condition).

Movie S1. Notch signaling oscillations in wild-type PSM explants cultured with 2.0 mM glucose or galactose. Real-time imaging of LuVeLu was performed to visualize Notch signaling dynamics.

Movie S2. Rescuing the segmentation clock oscillations with a sub-minimal amount of glucose. Real-time imaging of LuVeLu was performed to visualize Notch signaling dynamics.

Movie S3. Metabolic entrainment of Notch signaling oscillations by periodic modulation of glycolytic flux. Glucose concentration of the culture medium was periodically changed from 2.0 mM to 0.5 mM every 140-min. Real-time imaging of LuVeLu was performed to visualize Notch signaling dynamics.

Movie S4. Metabolic entrainment of Notch signaling oscillations by periodic pulses of FBP. Explants were subjected to 20 mM of FBP every 140-min. Real-time imaging of LuVeLu was performed to visualize Notch signaling dynamics.

Movie S5. Metabolic entrainment of Notch signaling oscillations by periodic pulses of pyruvate. Explants were subjected to 20 mM of pyruvate every 140-min. Real-time imaging of LuVeLu was performed to visualize Notch signaling dynamics.

Movie S6. Metabolic entrainment of Notch signaling oscillations by periodic pulses of 2,5-AM. Explants were subjected to 20 mM of 2,5-AM every 140-min. Real-time imaging of LuVeLu was performed to visualize Notch signaling dynamics.

Movie S7. Metabolic entrainment of Wnt signaling oscillations by periodic pulses of FBP. Explants were subjected to 20 mM of FBP every 140-min. Real-time imaging of Axin2-Achilles knock-in reporter was performed to visualize Wnt signaling dynamics.

Movie S8. Notch signaling oscillations in control and cytoPFKFB3 explants cultured with 2.0 mM glucose. Real-time imaging of LuVeLu was performed to visualize Notch signaling dynamics.

Movie S9. Wnt signaling oscillations in control and cytoPFKFB3 explants cultured with 2.0 mM glucose. Real-time imaging of Axin2-Achilles knock-in reporter was performed to visualize Wnt signaling dynamics.
